# Transcriptome plasticity underlying plant root colonization and insect invasion by *Pseudomonas protegens*

**DOI:** 10.1038/s41396-020-0729-9

**Published:** 2020-09-02

**Authors:** Pilar Vesga, Pascale Flury, Jordan Vacheron, Christoph Keel, Daniel Croll, Monika Maurhofer

**Affiliations:** 1 Plant Pathology, Institute of Integrative Biology, ETH Zürich, Zürich, Switzerland; 2 Department of Fundamental Microbiology, University of Lausanne, Lausanne, Switzerland; 3 Department of Crop Sciences, Research Institute of Organic Agriculture FiBL, Frick, Switzerland; 4 Laboratory of Evolutionary Genetics, Institute of Biology, University of Neuchâtel, Neuchâtel, Switzerland

**Keywords:** Bacterial pathogenesis, Bacterial host response, Bacterial genomics

## Abstract

*Pseudomonas protegens* shows a high degree of lifestyle plasticity since it can establish both plant-beneficial and insect-pathogenic interactions. While *P. protegens* protects plants against soilborne pathogens, it can also invade insects when orally ingested leading to the death of susceptible pest insects. The mechanism whereby pseudomonads effectively switch between lifestyles, plant-beneficial or insecticidal, and the specific factors enabling plant or insect colonization are poorly understood. We generated a large-scale transcriptomics dataset of the model *P. protegens* strain CHA0 which includes data from the colonization of wheat roots, the gut of *Plutella xylostella* after oral uptake and the *Galleria mellonella* hemolymph after injection. We identified extensive plasticity in transcriptomic profiles depending on the environment and specific factors associated to different hosts or different stages of insect infection. Specifically, motor-activity and Reb toxin-related genes were highly expressed on wheat roots but showed low expression within insects, while certain antimicrobial compounds (pyoluteorin), exoenzymes (a chitinase and a polyphosphate kinase), and a transposase exhibited insect-specific expression. We further identified two-partner secretion systems as novel factors contributing to pest insect invasion. Finally, we use genus-wide comparative genomics to retrace the evolutionary origins of cross-kingdom colonization.

## Introduction

*Pseudomonas* is a highly versatile genus that comprises bacteria living in diverse environments and that colonizes an ecologically broad range of hosts [1–3]. Some pseudomonads are pathogens of plants or animals such as fish, insects, or mammals [3, 4]. Members of the *Pseudomonas fluorescens* group [1, 2] are known plant growth-promoting rhizobacteria that stimulate plant growth, induce systemic resistance against foliar diseases and control soilborne fungal pathogens [5–8]. Due to these characteristics, several *Pseudomonas*-based biocontrol products are currently deployed to control fungal and bacterial diseases [9, 10]. Microbial-based methods for pest control will be crucial in future agricultural practices because an increasing number of chemical fungicides and insecticides is already or will likely be banned due to environmental and health concerns [11–13]. Within the *P. fluorescens* group, the two species *Pseudomonas protegens* and *Pseudomonas chlororaphis* are particularly interesting for plant protection applications because, unlike other biocontrol pseudomonads, they are crop plant colonizers with antifungal activity and pest insect colonizers with insecticidal activity [14–16].

*P. protegens* and *P. chlororaphis* colonize the insect gut after oral intake and transmigrate into the hemolymph, causing systemic infections and the eventual death of several Lepidoptera, Diptera, Coleoptera, and Hemiptera insect species [15–23]. The *P. fluorescens* subgroup [2] harbors insecticidal strains with lower pathogenicity than the *P. protegens*/*P*. *chlororaphis* species [14, 16, 18, 22]. In addition, *Pseudomonas aeruginosa* and *Pseudomonas entomophila* are also able to infect and kill different insect species, through different mechanisms [24, 25].

The first insecticidal trait discovered was the Fit toxin [17] typically produced by strains belonging to the *P. protegens* and *P. chlororaphis* species [16]. The contribution of this protein toxin to oral and systemic insecticidal activity and its tight insect host-dependent regulation were studied in some detail in *P. protegens* type strain CHA0 [15, 20, 26, 27]. Fit toxin production only partially explains the insecticidal capabilities of these bacteria as *fit* deletion mutants retain some toxicity [15, 20]. Studies on *P. protegens* CHA0 and other *P. protegens*/*P. chlororaphis* strains related insecticidal activity and host persistence to additional factors, including type VI secretion components [28], chitinase and phospholipase C [16], hydrogen cyanide [29], the cyclic lipopeptide orfamide [29, 30], the toxins rhizoxin [31] and IPD072Aa [32], and specific lipopolysaccharide O-antigens [33]. *P. protegens*/*P. chlororaphis* strains can also cause nonlethal infections [18, 22, 23, 31]. Even if the infection does not kill the insect after oral uptake, strains such as CHA0 can persist until pupal and imago stages, thus affecting the insect development as shown for *Delia radicum*, *Plutella xylostella* and *Pieris brassicae*, and be transmitted to new host plants by *D. radicum* [23]. Therefore, the ability of *P. protegens* to colonize cross-kingdom insect and plant hosts is impressively demonstrated by work on the model strain CHA0. However, it remains largely unknown what specific traits underlie cross-kingdom host colonization and how plastic responses including transcriptional remodeling contribute to switching between hosts.

We analyzed the transcriptome of *P. protegens* CHA0 during the colonization of plant roots, as well as from different compartments of insect hosts, specifically the hemolymph and gut, representing different stages of infection. We provide the first evidence for transcriptome remodeling underlying switches between insect pathogenic and plant beneficial lifestyles. We showed that CHA0 uses a host-specific set of tools for roots and for different insect compartments. Finally, we use genus-wide comparative genomics to retrace the evolutionary origins of cross-kingdom host colonization.

## Material and methods

### Preparation of *P. protegens* CHA0 samples from different hosts and environments

For each host/environment four independent replicate samples were prepared. From all samples an aliquot was used for assessment of bacterial numbers by plating serial dilutions onto King’s B+++ agar (see Supplementary Methods) [34, 35]. The remaining samples were immediately frozen in liquid nitrogen.

#### Grace’s insect medium (GIM) and lysogeny broth (LB)

*P. protegens* CHA0 was grown on KB+++ agar for two days. Single colonies were transferred to LB [36] or GIM (Sigma Aldrich, MO, USA) and grown to OD_600_ = 1.74–1.86 (~1.5 × 10^9^ cells/ml) at 24 °C while shaking at 180 rpm. Four milliliters of cultures were centrifuged (7500 rpm) and pellets used for RNA extractions.

#### Wheat roots

Root colonization assays were performed as described in de Werra et al. [37] and further explained in Supplementary Methods. Briefly, pre-germinated seeds of spring wheat, variety Rubli, were inoculated with 1 ml of a suspension containing 10^8^ CHA0 cells/ml and placed into seed germination pouches. Plants were grown at 22 °C and 60% humidity with a 16/8 h day (270 μmol m^−2^ s^−1^)/night cycle. After 1 week, roots of 99 wheat plants per replicate were harvested, shaken in 0.9% NaCl, the resulting suspensions were centrifuged and pellets containing bacteria were pooled for RNA extraction.

#### *P. xylostella* gut (oral infection)

*P. xylostella* eggs were kept before and during the experiment at 25 °C, 60% relative humidity and a 16/8 h day/night cycle with 162 μmol m^−2^ s^−1^. Second instar *P. xylostella* were fed with pellets of artificial diet spiked with 4 × 10^6^ CHA0 cells or NaCl 0.9% (control) as previously described by Flury et al. [29] and further explained in Supplementary Methods. For each replicate 120 treated alive larvae were collected 24 or 36 h post-feeding, surface disinfected and homogenized and homogenates were pooled. Sixty-three to sixty-five larvae per treatment were used for assessing survival over time. In addition, the resident cultivable microflora of untreated insects was assessed by growing the extracts on LB media plates at 18, 24, and 37 °C for 1 week.

#### *G. mellonella* hemolymph (hemocoel injection)

Seventh instar *G. mellonella* larvae were injected with 2 × 10^3^ CHA0 cells or 0.9% NaCl solution as previously described by Flury et al. [29] and further explained in Supplementary Methods. After 24 h, 55 alive non-melanized larvae per replicate were surface disinfected, one leg was cut and the hemolymph collected. Thirty to fifty larvae per treatment were used to assess survival.

### RNA extraction and sequencing

The range of total numbers of CHA0 cells used for sequencing were: LB, 5.6–5.9 × 10^9^; GIM, 5.5–5.9 × 10^9^; wheat, 5 × 10^8^–2 × 10^10^; *P*. *xylostella* 24 h, 2.8 × 10^6^–4.1 × 10^7^; *P*. *xylostella* 36 h, 2.4 × 10^6^–1.6 × 10^8^; and *G. mellonella*, 8.2 × 10^7^–2.2 × 10^9^ cells. RNA from insect and media samples was extracted using the GENEzol Reagent (Geneaid International, Taiwan) and from wheat root samples using the RNeasy Plant Mini Kit^TM^ (Qiagen, Germany) without bead-ruption. All extracts were treated with DNase from the RNeasy Mini Kit^TM^ (Qiagen, Germany). RNA quality was assessed using the 2200 Tapestation (Agilent, CA, USA) and Nanodrop 2000 (Thermo Scientific, MA, USA). For details, see Supplementary Table S1. Libraries were prepared using the TruSeq Stranded Total RNA Library Prep Kit. The RiboZero Bacteria kit was used for medium samples, RiboZero Epidemiology for insect samples and Ribozero Plant Root for wheat samples to remove bacterial, insect and plant rRNA, respectively (Illumina, CA, USA). Samples were sequenced using Illumina NextSeq500 single-end 81 bp sequencing (Illumina, CA, USA) at the Genomics Facility BSSE in Basel, Switzerland.

### Bioinformatics analysis

#### Read trimming, alignment, and normalization

Raw reads were quality trimmed and filtered for adapter contamination with Trimmomatic 0.36 [38]. Processed reads were aligned against the *P. protegens* CHA0 genome (NCBI entry number LS999205.1, [39]) using STAR 2.3.5a [40]. Mapped reads were counted with featurecounts 1.5.3 [41] using the stranded option and normalized with the TMM (trimmed mean of *M* values) method [42] of the edgeR 3.26.6 package [43] in R 3.6.0 (www.r-project.org). Genes with <1 count per million (CPM) in the four biological replicates were discarded. Normalized CPM were used for: (1) a multidimensional scaling analysis; (2) a transcriptome profile analysis with the K-means algorithm. The optimal number of clusters was assessed with the sum of square error method, which showed that six clusters can optimally predict the different transcription patterns; (3) a differential gene expression (DGE) analysis following the general linear model from edgeR package [43, 44]. The differentially expressed genes were determined using “glmFit” function with a Benjamin–Hochberg FDR correction for false positives; (4) Log10 transformation and heatmap generation with heatmap.2 package (https://CRAN.R-project.org/package=gplots).

Finally, predicted *P. protegens* CHA0 coding genes were assigned to Gene Ontology (GO) terms using InterproScan 5.27-66.0 [45]. A GO database was generated with GO.db 3.8.2 package [46]. All DGE genes and transcription profile main clusters genes were used in GO enrichment with GOstats 1.7.4 package in R [47].

#### Ortholog analysis

Whole protein sequences of 97 *Pseudomonas* species (Supplementary Table S2) were compared using OrthoFinder 2.3.3 [48] in an orthologue protein analysis with the default settings. Results were then filtered for chosen categories of proteins. The tree output was represented using FigTree 1.4.4 (http://tree.bio.ed.ac.uk/).

The detailed RNA-seq Script is placed in Supplementary Material 2.

### RT-qPCR

In order to verify the RNA-seq results, the expression of selected genes *pap*, *chiD*, *pltA, tpsA2*, *tpsA4,* and the PPRCHA0_1961 IS3 transposase gene in the investigated environments/hosts were quantified using RT-qPCR as described in Supplementary Methods, and Supplementary Tables S3 and S4.

### Role of two-partner secretion (TPS) proteins

Domains of *tpsA1, tpsA2, tpsA3,* and *tpsA4* encoded proteins were predicted using the HMMER database (www.hmmer.org). The *tpsA* deletion mutants of CHA0 were constructed as described in Supplementary Methods and Supplementary Table S5 and tested for insecticidal activity in feeding assays of 32 or 64 *P. xylostella* larvae and in injection assays of 18 *G. mellonella* larvae as previously described.

### Statistics of insect assays

Survival data in the *P. xylostella* feeding and *G. mellonella* injection assays were evaluated using a Log-Rank test of the Survival package of R 3.6.0 (www.r-project.org) with a *p* value < 0.05.

## Results and discussion

We analyzed the transcriptomic plasticity enabling *P. protegens* CHA0 to colonize lepidopteran larvae and wheat roots. These constitute two very different ecological niches in which CHA0 is known to establish pathogenic and beneficial interactions, respectively. We used the CHA0 strain to inoculate wheat roots, feed *P. xylostella* larvae or inject the hemocoel of *G. mellonella* larvae.

**Fig. 1:**
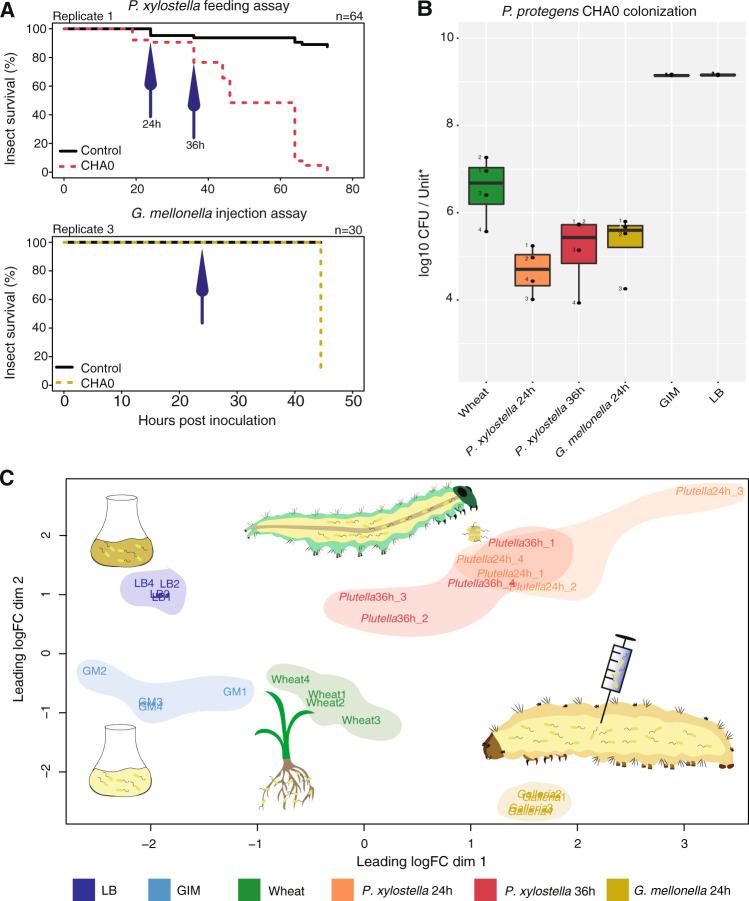
Toxicity (a), cell density (b), and MDS transcriptome analysis (c) for *Pseudomonas protegens* CHA0 colonizing different hosts and media. The “Wheat” samples correspond to wheat-roots 1 week after inoculation, “*P. xylostella* 24/36 h” to *Plutella xylostella* 24 and 36 h after oral infection; “*G. mellonella”* to *Galleria mellonella* hemolymph 24 h after hemocoel injection; “LB” to lysogeny broth, and “GIM” to Grace’s insect medium. **a** Survival of *P. xylostella* larvae after exposure to artificial diet pellets spiked with 4 × 10^6^ CHA0 cells (top) and of *G. mellonella* larvae upon injection of 2 × 10^3^ CHA0 cells (bottom). One representative experiment with 64 (*Plutella*) and 30 (*Galleria*) larvae is shown. Time points where insects were sampled for RNA extraction are indicated with arrows. **b** Bacterial densities at collection time points. Boxplots are created from four replicates per environment and show CFU per mg of root dry weight (wheat), CFU per mg of larvae (24/36 h), CFU per µl hemolymph (*G. mellonella*) and CFU per ml medium (GIM, LB). **c** Multidimensional Scaling (MDS) analysis was performed with four replicate CHA0 transcriptomes per environment. The different replicates of the different biological samples are numbered from 1 to 4.

We analyzed the transcriptome of CHA0 after 1 week on wheat roots, when bacteria had established population sizes of 10^6^–10^7^ CFU/mg dry root (Fig. 1b). At this time point, where roots elongate fast, we expect the transcriptome to represent the whole spectrum of bacterial root colonization with already established microcolonies on older roots and continuing growth towards the root tips. In addition, the absence of resident microflora from the soil or the plant, allowed us to observe transcriptomic variation exclusively in response to the plant.

*P. xylostella* was selected as model to study the progress of insect gut infection 24 and 36 h after feeding. Previous microscopy studies showed that, 1–2 days after feeding *P. xylostella* with treated pellets, CHA0 could only be found in the microvilli of the gut cells and, 3 days after feeding, the insect hemocoel was already heavily colonized by CHA0 [23]. In addition, *P. xylostella* larvae were shown to only harbor up to 100 cultivable CFU (including bacteria and fungi) of resident microorganisms per larvae at the moment of feeding with CHA0 (Supplementary Fig. 1c). In our study, at 24 h, the larvae showed no disease symptoms yet and were colonized by 10^4^–10^5^ CFU/mg larvae (Fig. 1a, b). At 36 h bacterial loads were ten times higher, some treated larvae started to die and the remaining were smaller and darker than non-treated larvae, indicating the start of bacterial transmigration from gut to hemocoel (Fig. 1a, b). Final mortality assessed in non-extracted *P. xylostella* was 90.6–98.4% (Fig. 1a and Supplementary Fig. S2). When CHA0 transmigrates into the hemocoel, it causes a systemic infection and eventually kills the insects.

Next, we wanted to understand the transcriptomic response in a pure hemolymph environment excluding transmigration factors. Therefore, we injected CHA0 directly into the hemocoel of the bigger insect model *G. mellonella* and extracted RNA 24 h later when the larvae were still alive, non-melanized and inoculant cell numbers had reached 10^5^ CFU/µl hemolymph (Fig. 1b). CHA0 killed 93.3–100% of *G. mellonella* larvae within 48 h (Fig. 1a and Supplementary Fig. S2). We used the *G. mellonella* injection model because to inject *P. xylostella* larvae without causing harm and to extract enough hemolymph for RNA sequencing without disrupting the gut is very difficult due to their small size.

In addition to CHA0 transcriptome remodeling on insect hosts, we analyzed the bacterial response to an insect-simulating culture medium without the influence of host immune responses i.e., GIM. Finally, the transcriptome of CHA0 was analyzed when growing in a rich culture medium (Lysogeny broth).

We obtained 10^8^–10^9^ reads from RNA Illumina NextSeq sequencing for each sample and quantified expression levels of CHA0 gene models (Supplementary Table S1). We performed a multidimensional scaling analysis to distinguish CHA0 transcriptomes according to the colonized host or culture condition (Fig. 1c). All transcriptomes were differentially separated except for the *P. xylostella* at 24 and 36 h due to the differences in infection progression across the biological samples. The first principal component axis of Fig. 1c separates the transcriptomes from culture media from those obtained from living hosts. This is probably due to the fact that bacteria need to actively colonize both wheat and insect hosts even though bacteria do not establish a pathogenic interaction on roots. This may explain the position of the wheat transcriptome between the culture media and the insect backgrounds. The second principal component separates *P. xylostella* gut and *G. mellonella* hemolymph samples (Fig. 1c). Some differences in gene expression between gut and hemolymph environments may be due to the different lepidopteran species used. However, CHA0 was shown to multiply in and kill larvae of different lepidopteran species following a similar pattern [15, 16, 20, 23, 26, 28]. Figure 1c also shows the transcriptome plasticity within the same insect species probably due to the implication of different bacterial factors at different phases in the infection.

### Pronounced differences between CHA0 transcriptomes during the colonization of wheat roots and insects

**Fig. 2:**
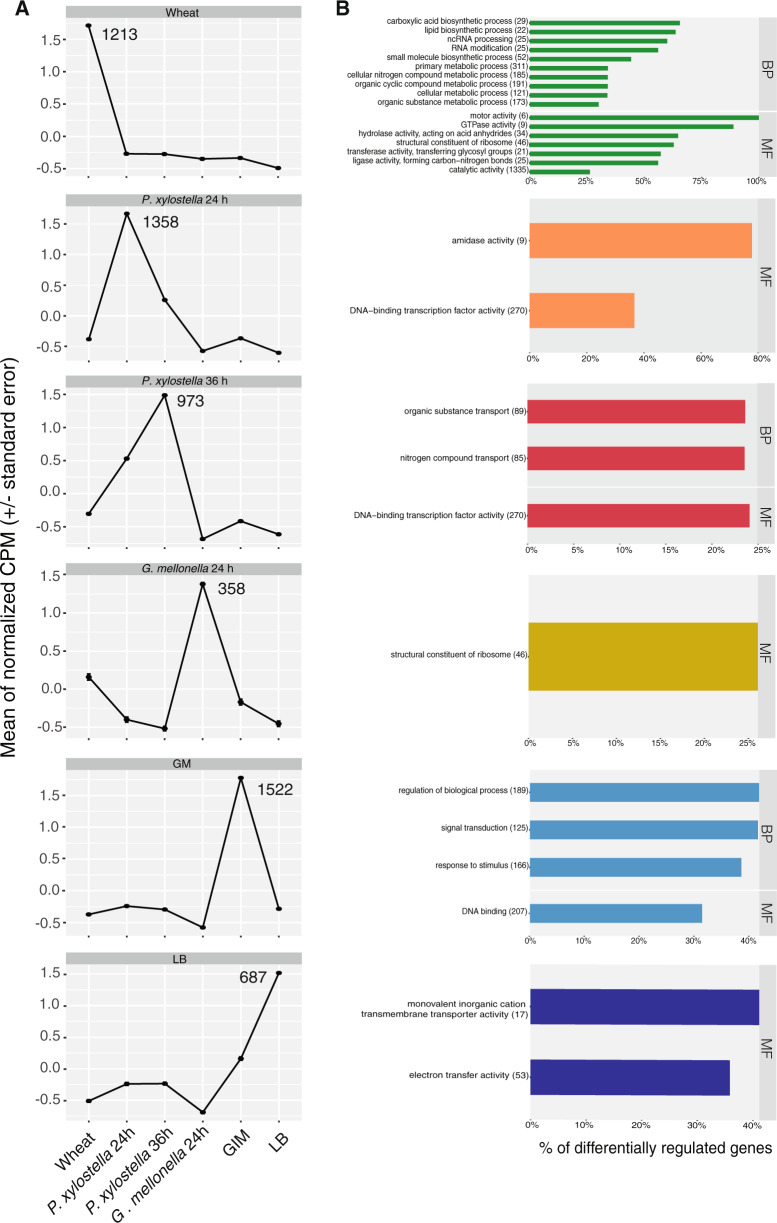
Transcription profiles of *Pseudomonas protegens* CHA0 during the colonization of different hosts or environments. **a** Normalized counts per million (CPM) of *P. protegens* CHA0 transcriptomes obtained using the K-means clustering method. Genes clustered in six different groups according to different hosts/media. Standard errors for four biological replicates show small variations among the genes included in each cluster. The number of genes that belong to the main cluster of each transcription profile are indicated. From each cluster, those genes with a gene ontology (GO) annotation were used in the enrichment analyses presented in **b**. **b** Gene ontology (GO) enrichment analysis of the specific genes of each transcription profile main cluster. Significant GO terms for the given set of genes are shown. The number of total genes related to a GO term present in the CHA0 genome is given between brackets and the indicated percentage shows how many of those have higher expression in the specific transcription profile (*p* value < 0.001). BP biological process, MF molecular function. Redundant GO terms were merged. The “Wheat” sample corresponds to wheat-roots 1 week after inoculation, “*P. xylostella* 24/36 h” to *Plutella xylostella* 24 and 36 h after oral infection; “*G. mellonella”* to *Galleria mellonella* hemolymph 24 h after hemocoel injection, “LB” to lysogeny broth; “GIM” to Grace’s insect medium.

The clustering analysis revealed distinct expression profiles for each of the hosts and culture condition (Fig. 2a). This implies that the CHA0 transcriptome changes drastically according to the colonized plant or insect host. We also monitored pronounced differences between the *P. xylostella* gut and the *G. mellonella* hemolymph, which indicates that a different set of genes is active in different insect compartments. But we cannot exclude that some of the observed differences might be due to the use of two different insect species.

**Fig. 3:**
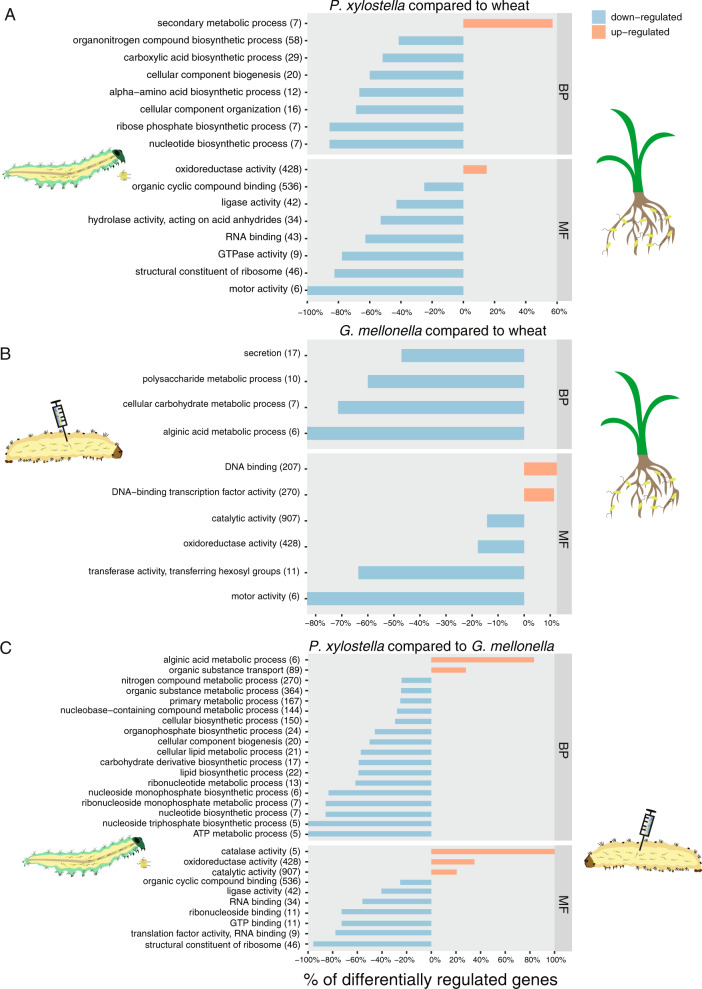
Comparisons of transcriptomes of *Pseudomonas protegens* CHA0 between different hosts (a, b) or between different insect compartments (c). CHA0 transcriptomes colonizing different hosts were analyzed by the general linear model pipeline of edgeR package in R. Transcriptomes derived from different hosts were compared pairwise; the reference is always the host shown in the right side of the figure. Total differentially expressed genes for each comparison for each condition were subjected to a GO enrichment analysis. Significant GO terms for the given set of genes are shown. Total genes related to a GO term present in the CHA0 genome are given between brackets and the indicated percentage shows how many of those are differentially expressed in each comparison (*p* value < 0.001). **a** *P. xylostella* vs. wheat roots, **b** *G. mellonella* hemolymph vs. wheat roots, **c** *P. xylostella* vs. *G. mellonella* hemolymph. BP biological process, MF molecular function. The “Wheat” sample corresponds to wheat-roots 1 week after inoculation, “*P. xylostella* 24/36 h” to *Plutella xylostella* 24 and 36 h after oral infection; “*G. mellonella*” to *Galleria mellonella* hemolymph 24 h after hemocoel injection.

We found that general metabolic processes and genes related to organic compound biosynthesis were upregulated on wheat roots (Figs. 2b and 3a). Genes related to nucleotide and protein synthesis were downregulated during insect colonization compared to wheat roots (Fig. 3a). This might indicate that at the chosen time points the bacterium was more metabolically active on the roots e.g., colonizing growing root zones, whereas bacterial growth may have been restricted in the gut. Though, our study reflects only specific moments in time and we cannot generally conclude that proliferation on the roots was higher than proliferation in the gut. Observed differences in gene expression could be caused by host-derived factors or by differences in growth rate or population sizes. This implies that the CHA0 transcriptome changes significantly depending on the colonized plant or insect. We also found pronounced differences between *P. xylostella* gut and *G. mellonella* hemolymph suggesting that a different set of genes is used for different insect compartments. However, the use of two insect species may influence on the observed differences.

**Fig. 4:**
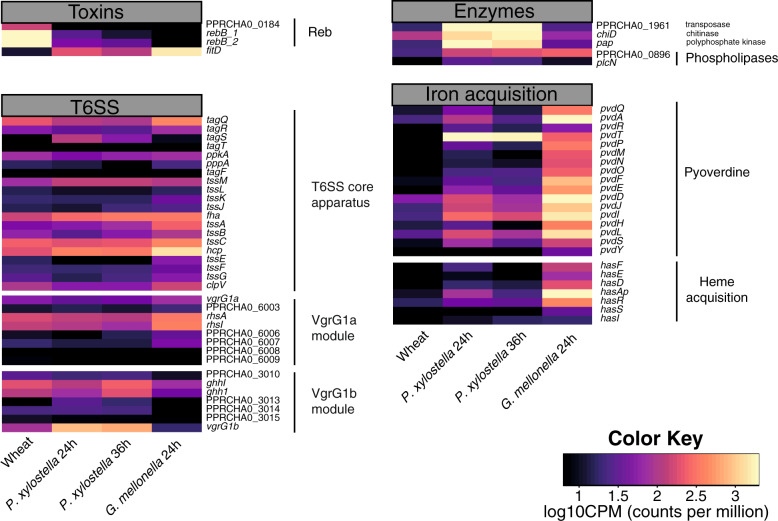
Heatmap showing the normalized reads (counts per million) for genes related to toxins, type VI secretion system (T6SS), specific enzymes, and iron acquisition in *Pseudomonas protegens* CHA0 colonizing different hosts. Black indicates low expression (less than 10 counts per million reads) and yellow indicates high expression (more than 10^3^ counts per million reads). The “Wheat” sample corresponds to wheat-roots 1 week after inoculation, “*P. xylostella* 24/36 h” to *Plutella xylostella* 24 and 36 h after oral infection; “*G. mellonella”* to *Galleria mellonella* hemolymph 24 h after hemocoel injection.

To successfully reach root zones where exudates are released and to outcompete other organisms, the bacteria need to actively move [49–51]. Motor activity-related genes were expressed under all conditions but especially upregulated on wheat roots as shown by clustering, DGE and heatmap analyses (Figs. 2b, 3a, b and Supplementary Fig. S3). This confirms the relevance of flagella for wheat root colonization. Interestingly, the *reb* genes required for R-body synthesis were upregulated on wheat roots and repressed in both insect compartments (Fig. 4), which supports the lack of differences in insecticidal activity between a Δ*rebB1* mutant and wildtype CHA0 in oral and injection assays [16]. R-bodies disrupt membranes and deliver toxins in several bacterial genera including *Pseudomonas*. In *Azorhizobium caulinodans*, the R-bodies have also been related to *Paramecium* killing and cell-disruption in the legume *Sesbania rostrata* [52–54].

### Specific activities in the insect gut are related to defense against the host immune system and to competition

The gut is a challenging environment for exogenous bacteria as they have to compete with the resident microflora and to overcome the insect immune response e.g., antimicrobial peptides (AMPs) and reactive oxygen and nitrogen species (ROS and RNS) [55]. The *P. xylostella* expression profiles reveal upregulation of several genes, which might help CHA0 to cope with these menaces in the gut. The *P. xylostella* 24 h transcription profile main cluster harbors most of the genes related to amidase activity (Fig. 2). Amidases cleave proteoglycans that trigger host immune response thereby helping bacteria to avoid recognition [55–57]. We suggest a similar mechanism in our model. At 36 h after feeding, we found that genes related to the transport of nitrogen and organic substances were specifically upregulated (Fig. 2b, *P. xylostella* 36 h). This might be related to the use of nitrogenous compounds emerging from the interaction of ROS and RNS during the insect immune response [58]. The combined evidence from the *P. xylostella* 24/36 h transcriptomic responses compared to wheat root transcriptomes showed upregulation of coding genes related to oxidoreductase activity proteins (Fig. 3a). This could be related to the bacterial defense against ROS produced by the insect host. Among the most upregulated genes in the *P. xylostella*-wheat comparison, were *pap* and PPRCHA0_1961 encoding a polyphosphate kinase (PPK) and a predicted “copy-paste” transposase, respectively (Fig. 4 and Supplementary Table 6). PPKs have been related to motility, quorum sensing, biofilm formation, and virulence of *P. aeruginosa* and regulation of stress response in *Campylobacter jejuni* [59–63]. We hypothesize that *pap* might play a similar role in CHA0 insect pathogenesis. The PPRCHA0_1961-encoded transposase might perform genomic rearrangements which are important for bacterial adaptation and pathogenesis as shown for *P. aeruginosa* with another transposable element family [64, 65].

The insect immune response also triggers the production of AMPs that kill invasive bacteria [55]. The dominant short O-antigenic polysaccharide (O-PS) encoded by the OSA cluster confers resistance to insect AMPs in CHA0 [33]. This supports invasion competences through oral and systemic insecticidal activity of the bacterium. Of the four CHA0 O-PS clusters [33], OSA and OBC3 (which encodes the major long O-antigen of CHA0) were expressed in all our backgrounds. Interestingly, OBC1 and OBC2 were only expressed in the *P. xylostella* gut (Supplementary Fig. S3). It remains unknown whether OBC1 and OBC2 might also play a role in avoiding resistance to or recognition by the insect immune system.

In order to persist, attach and breach the gut epithelium, CHA0 must successfully compete against the resident gut bacteria. CHA0 competes in the rhizosphere by producing a variety of antimicrobial compounds, which contribute to rhizosphere competence and the suppression of soilborne pathogens [5]. Among the tested hosts, all of the biosynthetic genes involved in the production of the broad-spectrum antimicrobials hydrogen cyanide, 2,4-diacetylphloroglucinol, and pyrrolnitrine were expressed in *P. xylostella* and expressed at low levels on wheat roots (with the exception of 2,4-diactetylphloroglucinol which was highest expressed on roots), but were not expressed in the *G. mellonella* hemolymph. Although the expression of hydrogen cyanide is low in our study, it has been previously shown to influence insect survival when CHA0 is injected into the *G. mellonella* hemocoel [29]. Although the expression of pyoluteorin biosynthetic genes was not detected on the roots of any tested plants [37], they were expressed in *P. xylostella* (Supplementary Fig. S3). Hence, pyoluteorin might play a more important role in persistence in insect hosts than on plant roots. Combined, our results support that antimicrobials are not only used for competition against microorganisms in the rhizosphere but also during colonization of an insect. We think that the expression of antimicrobials was mainly modulated by the insect background and less by the presence of resident microorganisms in the *Plutella* gut because the analysis of control larvae revealed a very low microbial background cultivable on LB (Supplementary Fig. S1). Likewise, CHA0 expresses antimicrobials on roots, even in the absence of other microorganisms [37].

The type VI secretion system (T6SS) has been related to pathogenicity and bacterial competition in the insect gut. In CHA0, the *vgrG1a* and *vgrG1b* genes encoding distinct T6SS spikes and *rhsA* and *ggh1* encoding respective associated effectors with predicted nuclease activity were demonstrated to contribute to invading *P. brassicae*. These genes play a role in the ability of CHA0 to compete with the gut microflora and impact on its composition [28]. In our study, these genes were expressed in both insect models (Fig. 4). This underlies the importance of these T6SS components during competitive host invasion [66–68]. Interestingly, the expression of *vgrG1b* and its associated effector gene *ggh1* was higher in the *P. xylostella* gut than in the *G. mellonella* hemolymph. But we found the opposite for *vgrG1a* and *rhsA* (Fig. 4) indicating that T6SS-mediated competition is important not only in the gut but also at a later infection stage in the hemolymph.

Expression patterns of *pap*, *chiD*, *pltA,* and the PPRCHA0_1961 IS3 transposase analyzed by qPCR showed the same tendencies as in the RNA-seq with significant differences between environments (*p* < 0.05) (Supplementary Fig. S4).

### Functions underlying transmigration from the gut lumen into the hemocoel

In order to transmigrate from the gut to the hemolymph CHA0 needs to overcome several barriers such as the peritrophic matrix and the gut epithelium. Based on the established expression profiles, we propose the following model of the transmigration process.

Orfamide A, the chitinase and the phospholipase C encoded by *ofaABC*, *chiD,* and *plcN,* respectively, showed the highest expression in *P. xylostella* when compared to the other environments (Fig. 4 and Supplementary Fig. S3). In previous studies, CHA0 mutants lacking any of these genes had reduced activity in oral *P. xylostella* feeding assays, but not upon injection into *G. mellonella* hemolymph [16, 29]. This suggests that *ofaABC*, *chiD,* and *plcN* are important in the gut infection/transmigration phase. Orfamide A and chitinases were shown to be important in insect pathogenesis even though the exact mechanism remains to be elucidated [29–31]. We hypothesize that orfamide A might be important for adsorption to the peritrophic matrix that lines the gut epithelium and the chitinase might create a passage through this chitinous membrane into the mucus layer. The mammal lung and gut mucus layers are rich in phosphatidylcholine and phosphatidylserine, two major components of eukaryotic membranes [69]. Phosphatidylserine and phosphatidylcholine are cleaved by PlcN [70] and used as a nutrient source in *P. aeruginosa* [71, 72]. Therefore, we propose that the nonhemolytic PlcN phospholipase might release phospholipids from the gut mucus and weaken the enterocyte membranes in insects. At a later stage of infection, *Pseudomonas* probably use exopolysaccharides to attach to the epithelium as described in chronic lung infections by *P. aeruginosa* [73–76] before they transmigrate to the hemocoel. We identified the protein functions associated to alginic acid metabolism and the transport of organic compounds being differentially regulated when comparing *P. xylostella* to *G. mellonella* (Fig. 3c) supporting our model. The CHA0 exopolysaccharide gene clusters *alg*, *psl*, *pga,* and *pel* were barely expressed in the *G. mellonella* hemolymph but were upregulated on wheat roots and in *P. xylostella*. This upregulation could play a role in attachment to the insect gut surface (Supplementary Fig. S3).

Once the bacteria have passed through the peritrophic matrix and are attached to the gut, they need to disrupt the epithelial cells to create a passage to the hemocoel. Based on our transcriptomic evidence, we found that TPS systems may be involved in this process. TPS of Gram-negative bacteria consist of a B transporter and an A effector protein, e.g., an adhesin, hemolysin, or exolysin [77]. TPS secreted toxins were identified in different Gram-negative bacteria e.g., in *P. aeruginosa*, *Serratia marcescens*, *Proteus mirabilis*, *Haemophilus influenza*, and entomopathogenic bacteria such as *Photorhabdus luminescens* and *P. entomophila* [77–82]. TPS are related to cellular adhesion, pore formation, and competition across bacterial species [77, 83], host tissue damage [84], cell-junctions cleavage in *P. aeruginosa* PA7 [77, 82, 85], and macrophage pyroptosis in *P. aeruginosa* PA7, *P. entomophila* L48, *P. putida* KT2440, and *P. protegens* CHA0 by ExlA-like proteins [81].

**Fig. 5:**
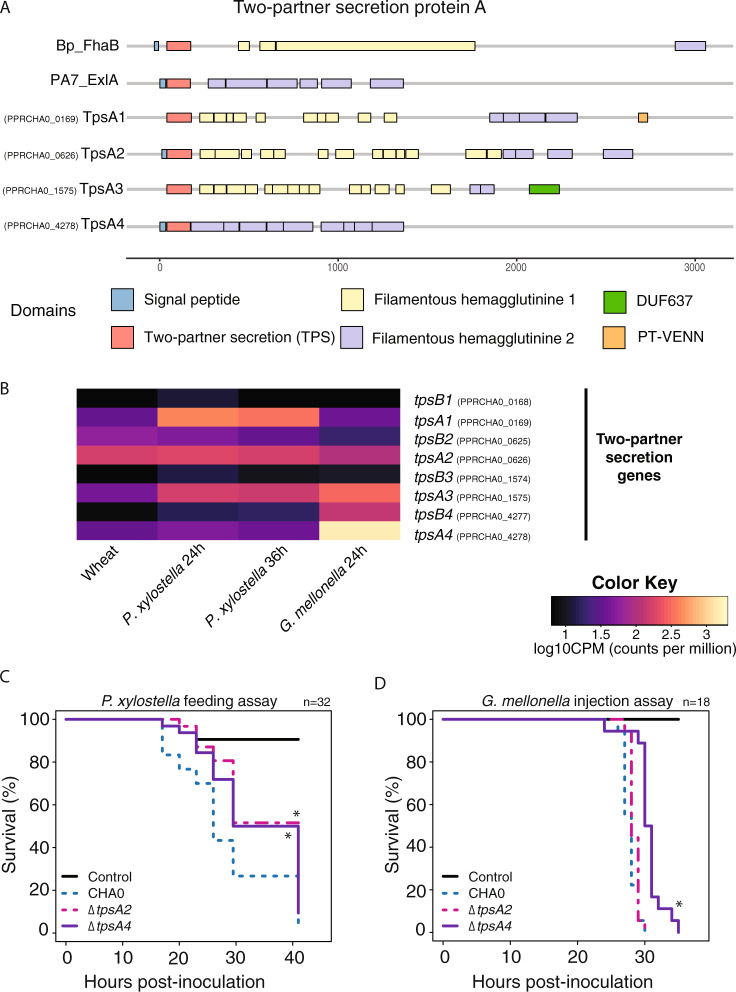
Two-partner secretion (TPS) systems in *Pseudomonas protegens* CHA0: domain analysis (a), expression profiles in relation to different hosts (b), and contribution to insecticidal activity (c). **a** Domain analysis of the secreted protein with HMMER database comparing *Bordetella pertussis* protein FhaB, *Pseudomonas aeruginosa* PA7 protein ExlA and *P. protegens* CHA0 proteins TpsA1, TpsA2, TpsA3, and TpsA4 (PPRCHA0_0168-169, PPRCHA0_0625-0626, PPRCHA0_1574-1575, and PPRCHA0_4277-4278, respectively). The signal peptide and TPS domains are used to interact with the transporter protein for membrane translocation; the filamentous hemagglutinin 1 attaches to the host-cell and the filamentous hemagglutinin 2 translocates the PT-VENN domain into the host; DUF637 is common to hemagglutinins but its function is still unclear [83]. **b** Heatmap showing the normalized expression values for genes related to the four complete two-partner secretion systems in *P. protegens* CHA0 colonizing different hosts. The “Wheat” sample corresponds to wheat-roots 1 week after inoculation, “*P. xylostella* 24/36 h” to *Plutella xylostella* 24 and 36 h after oral infection, “*G. mellonella”* to *Galleria mellonella* hemolymph 24 h after hemocoel injection. **c** Survival of *P. xylostella* larvae after exposure to artificial diet pellets spiked with 4 × 10^6^ cells of CHA0 wild type, or its *tpsA2* or *tpsA4* deletion mutants. Thirty-two 2nd instar larvae were used per bacterial strain. **d** Survival of *G. mellonella* larvae after injection of 2 × 10^3^ cells into the hemocoel. Eighteen 7th instar larvae were used per bacterial strain. One experiment of each is shown and two more *P. xylostella* feeding and one *G. mellonella* injection are shown in Supplementary Fig. S5. Asterisks indicate significant differences of mutants to the wildtype (log-rank test, *p* value < 0.05).

The CHA0 genome harbors four predicted complete TPS named *tpsBA1-4* encoding TpsBA1-4 (Fig. 5; PPRCHA0_0168-169, PPRCHA0_0625-0626, PPRCHA0_1574-1575, and PPRCHA0_4277-4278). TPSA1-4 effector proteins share between 39.3% and 59.5% amino acid identity with the ExlA protein of *P. aeruginosa* PA7 (Supplementary Table S7). Similarly to PA7, ExlA, and FhaB of *Bordetella pertussis*, CHA0 TpsA1-4 have a N-terminal TPS domain followed by several copies of filamentous-haemagglutinine domains (FLH1 and 2 domains, Fig. 5) [82, 83]. In addition, TpsA1 also has a pre-toxin VENN domain (Fig. 5a) commonly involved in cell–cell interactions [83, 86]. While the domain structure of TpsA4 is highly similar to PA7 ExlA, TpsA1-3 contain several predicted FLH1 repeats in addition to the FLH2 domains (Fig. 5).

The four *tpsA* genes showed very different expression profiles across the examined environments (Fig. 5b). While *tpsA2* was equally expressed in all examined hosts, the other three variants were upregulated in insects when compared to wheat roots. *tpsA3* was similarly expressed in both insect models, in comparison to *tpsA1* which was upregulated during *P. xylostella* infection and *tpsA4* which was highly upregulated in *G. mellonella* hemolymph. Interestingly, *tpsA4* was among the 20 most upregulated genes in *G. mellonella* when compared to *P. xylostella* (Supplementary Table S6).

In order to assess the importance of TpsA toxins for insect infection, we tested *tpsA* knockout mutants in feeding assays with *P. xylostella* and in injection assays with *G. mellonella*. The deletion of *tpsA4*, which was highly expressed in an insect background but not on wheat roots, resulted in significantly reduced insecticidal activity in two out of three feeding experiments. A deletion mutant of *tpsA2*, which was expressed in all tested hosts, led to significantly reduced insecticidal activity in one out of three feeding experiments (*p* < 0.05) (Fig. 5c and Supplementary Fig. S5a, b). In addition, a *tpsA4* deletion mutant showed significantly slower mortality when injected into the *G. mellonella* hemocoel in two experiments (*p* < 0.05) (Fig. 5d and Supplementary Fig. S5c). Furthermore, we confirmed the expression patterns of *tpsA2* and *tpsA4* by qPCR showing the same tendencies as found in the transcriptomic analyses with significant differences between environments (*p* < 0.05) (Supplementary Fig. S4).

The functions of the CHA0 TPS-secreted toxins are still unknown, but these toxins may play similar roles as the *P. aeruginosa* toxin ExlA. TpsBA1-3 may be involved in gut colonization, adhesion to tissues, bacterial competition, and the disruption of the gut epithelium. The high expression of TpsBA4 in the hemolymph suggests that the protein could be involved in the defense against insect immune reactions e.g., by triggering hemocyte cell death. Our model of TPS interactions are supported by the findings that CHA0 mutants lacking t*psA4* are largely impaired in macrophage killing [81].

### The insect hemocoel is more permissive for rapid proliferation than the gut

During the first phase of gut infection, CHA0 shows limited growth and metabolism. In contrast, we found an upregulation of structural ribosome constituents and nucleic acid synthesis in the *G. mellonella* hemolymph (Figs. 2b and 3c). The pairwise comparison revealed a general upregulation of genes involved in proliferation and metabolic activities in the *G. mellonella* hemolymph compared to the *P. xylostella* gut (Fig. 3c). In contrast, oxidoreductase and catalase activity were downregulated compared to *P. xylostella* and wheat (Fig. 3c, b). These differences indicate that the hemolymph is a less stressful environment and that CHA0 has enough nutrients allowing rapid proliferation leading to the systemic infection and ultimately death of the insect. However, oxidoreductase functions were upregulated in the *G. mellonella* hemolymph when compared to the hemolymph mimicking Grace’s Medium (Supplementary Fig. S6 and Table S6). This may be related to defenses against insect immune responses. Furthermore, the bacteria are challenged by iron deprivation forcing a strong upregulation of pyoverdine synthesis and heme-acquisition related genes (Fig. 4 and Supplementary Table S6). Pathogenic bacteria need siderophores such as pyoverdine and heme-acquisition systems to acquire the essential iron from iron-binding proteins e.g., ferritin or transferrin in the gut lumen, the hemolymph, and the fat body. Also, the hemolymph pH is not acidic enough for the scarce free iron to be bioavailable for the bacteria [87–89]. However, mutants defective in pyoverdine production still show insecticidal activity [29], probably because the loss of pyoverdine is compensated by the production of other siderophores e.g., enantio-pyochelin during insect colonization [90, 91].

The Fit insect toxin substantially contributes to insect killing in systemic infections by CHA0 and other *P. protegens*/*P. chlororaphis* [15, 17, 20]. It possibly interferes with the activity of hemocytes as was shown for the related apoptotic toxin Mcf of *P. luminescens* [92, 93]. Previously, Fit was shown to be only produced in the insect hemolymph but not on plant roots using a mCherry-labeled FitD [20]. We can show now that *fitD* was among the 20 most upregulated genes in the *G. mellonella*-wheat comparison (Fig. 4 and Supplementary Table S6). Our study further shows that the maximal expression of *fitD* occurs in the hemolymph but that the upregulation is initiated when the bacteria colonize the insect gut (Fig. 4).

### Genus-wide comparative genomics to retrace the evolutionary origins of *Pseudomonas protegens* CHA0 pathogenicity factors

**Fig. 6:**
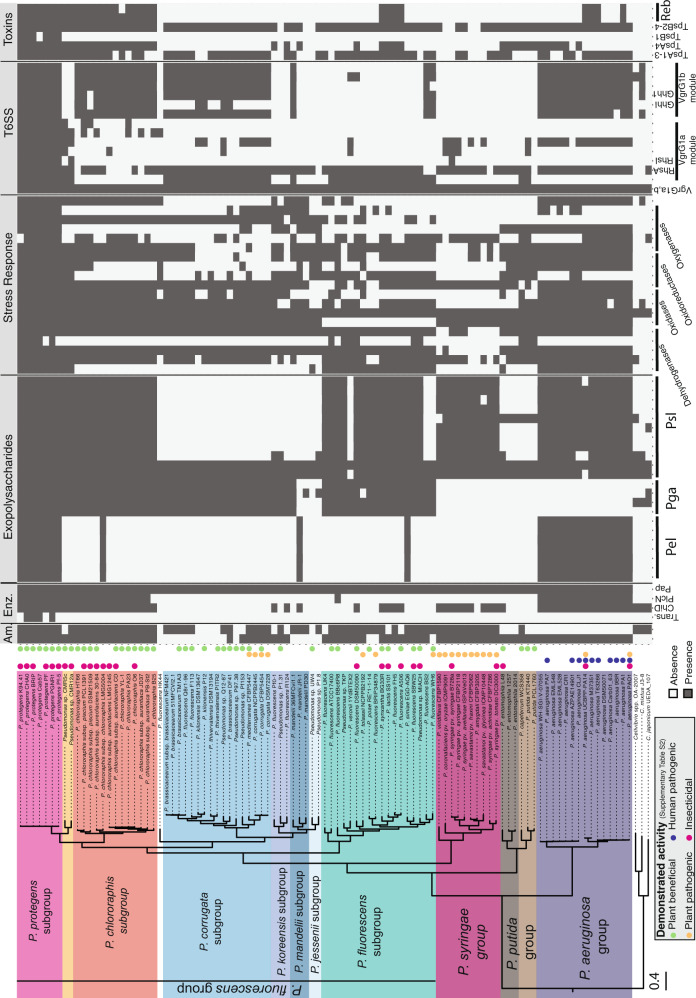
Orthologue protein analysis examining the presence of *Pseudomonas protegens* CHA0 factors associated with insect pathogenicity across different phylogenetic *Pseudomonas* groups. A comparison of the full in silico proteomes of 97 pseudomonads belonging to phylogenetic groups harboring insect pathogenic, human pathogenic, plant pathogenic, and plant beneficial strains (groups and subgroups as defined by Hesse et al. [2]) was performed and is shown in Supplementary Fig. S7. Here, we show the distribution of selected CHA0 traits investigated in this study in ecologically different *Pseudomonas* strains. Strains with described activity are marked in: pink for insecticidal activity (oral or injectable); dark-blue for human pathogenic activity; orange for plant pathogenic activity; green for plant-beneficial activity (references in Supplementary Table S2). Am. amidases, Enz. enzymes, Trans. transposase.

We compared the full protein sequence of CHA0 and 96 pseudomonads using ortholog analyses (Supplementary Fig. S7). We analyzed phylogenetic groups harboring strains showing plant beneficial interactions as well as plant-, insect-, and human pathogenic abilities (Supplementary Table S2). *P. protegens*/*P. chlororaphis* subgroups possess a set of specific traits absent in other *Pseudomonas* groups (Fig. 6 and Supplementary Fig. S7). We investigated whether CHA0 genes responding to lifestyle changes were common to other pathogenic and beneficial *Pseudomonas*. We focussed on genes with host or insect compartment specific expression that have emerged from this study and/or have been shown to contribute to insecticidal activity in earlier studies [16, 28] i.e., specific exoenzymes, exopolysaccharides, T6SS modules, and toxins.

Some genes with lifestyle specific expression patterns are distinct of the *P. protegens* subgroup including the transposase PPRCHA0_1961, Vgr1a elements e.g., the Rhsl effector [28] and TpsB1 (Fig. 6). This suggests that these genes were recently acquired by *P. protegens* or were lost in all other groups. We propose that some of these genes are specific for insect interactions. However, most functions in insecticidal activity are shared by the *P. protegens* and *P. chlororaphis* subgroups. Intriguingly, some of these traits are also present in the phylogenetically distant *P. aeruginosa* group [1, 2] harboring animal and human pathogens. Among these functions, we found a chitinase, the phospholipase PlcN, proteins related to the production of the exopolysaccharide Pel, T6SS components of the Vgr1A and Vgr1b modules, the Reb toxins or some proteins related to stress-response. The presence of these insect interaction-related CHA0 traits in very distant species such as *P. aeruginosa* (Fig. 6) suggests that either these are ancestral functions and were lost repeatedly during the evolution of *Pseudomonas* or that they have been independently acquired. Interestingly, the entomopathogen *P. entomophila* [24] lacks several of the CHA0 functions shared with the *aeruginosa* group including Pel, Psl, the Vgr1a, and b elements [28], and some stress-related proteins (Fig. 6). *P. chlororaphis* and *P. protegens* with the ability to colonize plant and insect environments seem to have a very distinct toolbox when compared to the rest of the analyzed species (Fig. 6 and Supplementary Fig. S7).

## Conclusions

**Fig. 7:**
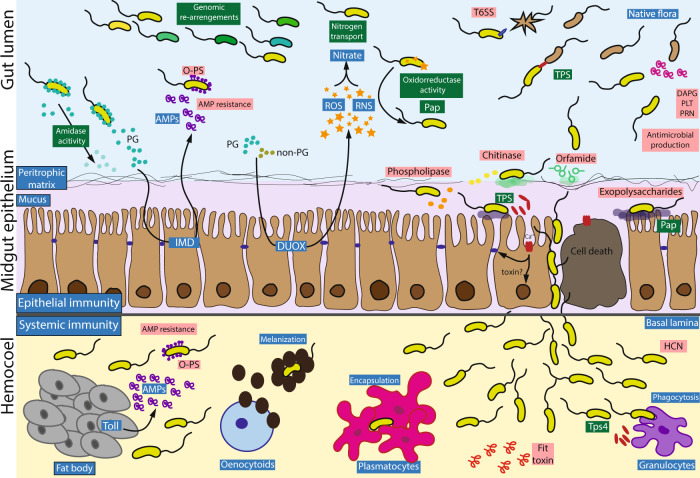
Proposed pathogenesis model of *P. protegens* CHA0 colonizing Lepidoptera insect pests after oral infection. In the proposed pathogenesis model, the insect immune response is marked in blue, CHA0 factors emerging from this study in dark green and factors shown to be involved in *P. protegens* CHA0 insecticidal activity in previous studies in pink. As described previously by Engel and Moran [55], in the event of a pathogen invasion, the insect will detect the presence of proteoglycans or other bacterial components that will trigger the immune response. The gut epithelium activates the production of reactive oxygen and nitrogen species (ROS and RNS) through the DUOX membrane oxidases and antimicrobial peptides (AMPs) through the IMD pathway. If the signal reaches the insect fat body, the Toll pathway will activate the production of AMPs as well [55, 94]. We hypothesize the following infection process: CHA0 is taken up by an insect feeding on plant colonized by the bacterium. In the gut, the bacterium faces the first line of the insect defense and has to compete with the resident microflora. Amidase activity degrading proteoglycan residues from the cell wall helps *P. protegens* CHA0 to avoid recognition by the immune system. The bacterium further uses oxidoreductases and the Pap protein to protect itself against reactive oxygen and nitrogen species. Nitrogen transporters might capture nitrogenous compounds resulting from the interaction of RNS and ROS. In order to better survive this adverse and stressful environment, it is possible that *P. protegens* CHA0 activates transposases for genomic rearrangements in order to increase its genomic variability. The bacterial cells can resist AMPs thanks to the O-polysaccharide conformation of its surface [33]. CHA0 also produces antimicrobial compounds as shown here and in Flury et al. [29] and it uses the type VI secretion system (T6SS) to fight the microflora of the insect or other ingested bacteria. For breaching the gut epithelium, we propose the following scenario: to adhere to the surface of the peritrophic matrix, CHA0 uses the cyclic lipopeptide surfactant orfamide A [29]. Then, the chitinase disrupts the chitinous peritrophic matrix [16]. *P. protegens* CHA0 may use the phospholipase PlcN to release nutrients from the mucus layer or to damage the epithelial cells [16, 95] and exopolysaccharides to establish in the epithelium [76]. Subsequently, the production of different two-partner secretion proteins (TPS) triggers host cell death and disrupts the cadherin junctions between epithelial cells. This will allow the bacteria to transmigrate into the hemocoel. Here CHA0 has to resist phagocytosis by granulocytes, encapsulation by plasmatocytes, melanin coating by oenocytoids and AMPs produced by the fat body [21, 96]. To fight the immune cells, CHA0 might use the FitD toxin [33], hydrogen cyanide (HCN) [29], and the TpsA proteins which, in combination with the bacterial multiplication, will finally lead to the death of the insect.

*P. protegens* and *P. chlororaphis* are bacterial species with multifaceted lifestyles as they can easily switch between plant and insect hosts. Our analyses of the *P. protegens* CHA0 transcriptomes across plant, insect and specific culture medium conditions significantly enhance our understanding of the shared and specific functions deployed across host-associated lifestyles. We have also shown how different functions are modulated over the course of an insect infection. Our results show that CHA0 deploys distinct toolsets to colonize plant-roots, the insect gut, and the insect hemocoel with specific expression in some environments (e.g., flagella on roots or the Fit toxin in the insect hemocoel). In contrast, we also discovered that antimicrobial metabolites, the T6SS, and exopolysaccharides serve as weapons or colonization factors across multiple environments. We also identified potential new factors involved in insect interactions of *P. protegens* CHA0 i.e., PPK and the transposase PPRCHA0_1961 and we demonstrated the contribution of TPS systems in insect pathogenicity. Based on the results presented here and our previous studies on insecticidal traits, we propose a comprehensive insect colonization and pathogenesis model for *P. protegens* CHA0 as summarized in Fig. 7.

We finally show that some key insect pathogenicity factors are conserved across *Pseudomonas* groups, while other factors are patchily distributed in *P. protegens* or *P. protegens*/*chlororaphi*s suggesting distinct evolutionary origins.

## Supplementary information

Supplementary Material 1

Supplemenetary Material 2

Supplementary Material 3

## Data Availability

The generated RNA-seq datasets were deposited on the NCBI Short Read Archive under the BioProject ID PRJNA595077.
