# Supplementary material for: Transcriptome plasticity underlying plant root colonization and insect invasion by Pseudomonas protegens
Source: ISME J. 2020 Sep 2;14(11):2766–82. doi: 10.1038/s41396-020-0729-9 (PMC7784888; doi:10.1038/s41396-020-0729-9)
Supplement: Supplementary file 1 — Supplementary Material 1 [file 41396_2020_729_MOESM1_ESM.docx]

**SUPPLEMENTARY INFORMATION**

**CONTENT**

**Supplementary Methods**

- Wheat root colonization assay
- *Plutella xylostella* feeding assay
- *Galleria mellonella* injection assay
- RT-qPCR
- Construction of *tpsA* mutants

**Supplementary Figures**

**Figure S1:** Colony forming units (bacteria and fungi-like) inhabiting *P. xylostella*.

**Figure S2.** Survival of *Plutella xylostella* and *Galleria mellonella* larvae after inoculation with *P. protegens* CHA0

**Figure S3.** Heatmap showing the normalized reads (counts per million) for genes related to exopolysaccharides, o-polyaccharides, flagellum and antimicrobials in *P. protegens* CHA0 colonizing different hosts

**Figure S4.** Confirmation of RNA-sequencing results by qPCR

**Figure S5.** Survival of *Plutella xylostella* and *Galleria mellonella* larvae when treated with

Δ*tpsA2* and Δ*tpsA4* mutants of *P. protegens* CHA0

**Figure S6.** Comparison of the CHA0 transcriptomes during colonization of the hemolymph of *Galleria mellonella* and Grace’s Insect medium

**Figure S7.** Orthologue comparison based on whole proteomes between different strains of *Pseudomonas*

**Supplementary Tables**

**Table S1.** Reads obtained from Illumina NextSeq sequencing of RNA extracted from different CHA0 inoculated host/environments

**Table S2**. List of *Pseudomonas* strains used in the orthologue analysis.

**Table S3:** Primers used for RT-qPCR

**Table S4.** Quantitative PCR conditions for LightCycler480 (Roche, Switzerland)

**Table S5.** Plasmids and primers used to create *tpsA2* and *tpsA4* deletion mutants

**Table S6** (separate Excel file): Differential gene expression in the comparison of two hosts/conditions

**Table S7:** Similarities of the predicted two-partner secretion A (TpsA) – like proteins of *P. protegens* CHA0 with related proteins in pathogenic bacteria.

**References**

**Supplementary Methods**

**Wheat-root colonization assay**

The assay described in de Werra et al., 2008 was used with some modifications. Spring wheat seeds of the variety Rubli (Delley Samen und Pflanzen AG, Delley, Switzerland) were surface disinfected for 30 min with 1.4% NaClO (vol/vol) and subsequently rinsed with autoclaved distilled H_2_O. Clean seeds were pre-germinated on 1.5% agar plates for two days and then transferred to CYG seed germination pouches (18 cm high by 16.5 cm wide, Mega International, West St. Paul, Mn, U.S.A.), three seeds per pouch. 1 ml of a suspension containing *P. protegens* CHA0 cells (OD_600_ ≈ 0.125 ≈ 10^8^ cell/ml) was inoculated onto the seeds. For each replicate 33 pouches with 3 seeds each were prepared. Plants were grown at 22 °C and 70% relative humidity with a 16/8 h day (270 μmol m^-2^s^-1^)/ night cycle. After one week, roots of 99 plants were harvested in batches of 9 plants, placed into 50 ml 0.9% NaCl and shaken at 400 rpm for 20 min. Pellets were pooled in 50 ml 0.9% NaCl. Two-hundred-fifty μl of the resulting suspension were used to assess bacterial colonization by plating dilution series onto King’ B [1] plates supplemented with three antibiotics (= KB+++): ampicillin, 40 μl ml^-1^; chloramphenicol, 13 μl ml^-1^; and cycloheximide, 100 μl ml^-1^ [2] The remaining suspension was centrifuged and the pellet containing bacteria frozen in liquid nitrogen and stored at -80 °C until RNA extraction. Four independent replicates were prepared.

***P. xylostella* feeding assay**

*P. xylostella* eggs were obtained from Syngenta Crop Protection AG (Stein, Switzerland). Insects were kept before and during the experiment at 25 °C, 60% humidity and 16h - 8h day (162 μmol m^-2^s^-1^)/night cycle. For the feeding assays, 185 second instar larvae were placed individually into wells of 128-well bioassay trays (Frontier Agricultural Sciences, Delaware, USA) to avoid cannibalism. Each well contained a wetted filter paper and a piece of diet pellet spiked with 10 μl NaCl 0.9% (control) or 10 μl of a suspension of *P. protegens* CHA0 cells (OD ≈ 0.5 ≈ 4 x 10^8^ cfu/pellet) resulting in 4 x 10^6^ cfu per pellet. The artificial diet used for the experiments was prepared by boiling 7.5 g of agar in 500 ml of distilled H_2_O for 1 min. Then 50 g of Adapta Bio-Dinkel cereal (Hero Baby, Switzerland), 1 effervescent vitamin pill (Santogen Gold, Switzerland), 15.5 g yeast extract (Difco, MI, USA), 7.5 g casaminoacids (Difco, MI, USA), 0.25 g cholesterol (Sigma Aldrich, MO, USA), 0.5 ml corn oil (Coop, Switzerland) were added and homogenized with a blender. The homogenate was poured in Petri dishes up to 2 mm of thickness and the pellets were further cut in 4 mm diameter pieces with a cork borer [3]. Sixty-three to sixty-five larvae per treatment were kept for assessing survival by repeated poking and the rest was prepared for RNA extraction as follows.

One-hundred-twenty larvae were collected 24 h and 36 h after feeding and surface disinfected by washing in ethanol 70% (20 s) and rinsing with sterile distilled H_2_O and 0.05% SDS (20 s) twice. The effectiveness of this method was evaluated in previous studies [4]. Portions of 30 surface disinfected larvae were homogenized in 1 ml NaCl 0.9% with a Polytron PT-DA 2112 blender (Kinematica, Littau, Switzerland). All homogenates were pooled. Two hundred fifty μl of the homogenate were used to assess bacterial colonization as described above. The remaining sample was centrifuged at 7500 rpm for 5 min, frozen in liquid N_2_ and preserved at -80°C until RNA extraction. Four replicate samples were prepared over time, only from batches (feeding assays) with final mortality higher than 90%.

***G. mellonella* injection assay**

Four replicates of last (seventh) instar *G. mellonella* larvae (Hebeisen Fishing, Zürich, Switzerland) were injected with 10 μl of a suspension containing 2 x 10^5^ *P. protegens* CHA0 cells or 10 μl of sterile 0.9% NaCl solution with a repetitive dispensing Tridak Stepper (Intertronic, Oxfordshire, UK). Groups of 10 larvae were kept together in a Petri dish at 24 °C in the dark until the end of the experiment. One part of the larvae, i.e. 30 in replicates 1 to 3 or 50 in replicate 4, were used to assess survival. Larvae were considered dead when they did not react anymore to repeated poking with a tip. The rest of the larvae (55) in each replicate were used for preparing samples for RNA extraction. After 24 h, these 55 larvae were surface disinfected as described for *P. xylostella* above. One leg was cut and the hemolymph was gently squeezed in an Eppendorf tube. For each larva, 1 μl of the hemolymph was used to assess bacterial colonization as described above. The harvested hemolymph from all 55 larvae was pooled, immediately frozen in liquid N_2_ and stored at -80 °C until RNA extraction. Four replicates were prepared over time.

**RT-qPCR**

RNA was transformed into cDNA by RevertAid First Strand cDNA Synthesis Kit^TM^ (Thermo Scientific, MA, USA) and used in a quantitative PCR reaction performed with Eva Green^TM^ (Biotium, CA, USA) in a LightCycler480 (Roche, Switzerland). Conditions and primers of the qPCR are reported in Supplementary Table S3 and S4. All four biological replicates were tested in triplicates. The amplification efficiency was further checked by the LinRegPCR software [5] to discard data points with an efficiency under 1.8. Each data point was adjusted with its individual efficiency. The expression of each gene was corrected with the 16S rRNA gene as reference and normalized to the expression of the respective gene on wheat roots according to Pfaffl model [6]. Wheat root was used as the calibrator sample to assess the differences between colonizing insects and roots. Mean expression differences were assessed by a Kruskal Wallis test and a pair-wise comparison (Dunn’s posthoc test, p<0.05) was performed comparing each background to wheat in R 3.6.0 ([www.r-project.org](http://www.r-project.org)).

**Construction of *tpsA* mutants.**

The *tpsA* deletions mutants of *P. protegens* CHA0 i.e. Δ*tpsA2* (PPRCHA0_0626) and Δ*tpsA4* (PPRCHA0_4278) were generated using the allelic replacement technique with the I-SceI system from the suicide vector pEMG [7] as described in Kupferschmied et al. [8] with the primer pairs listed in Supplementary Table S5.

**Supplementary Figures**


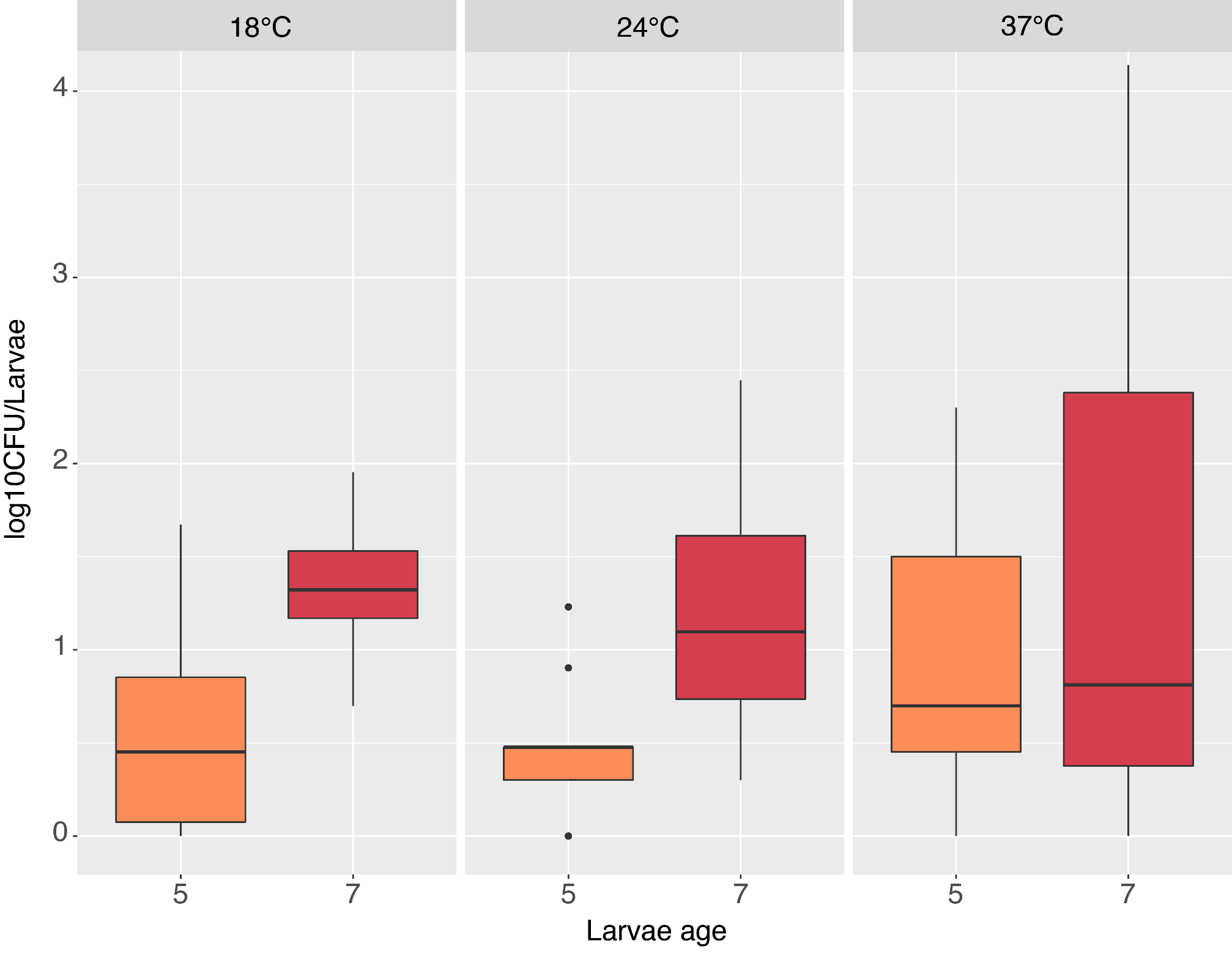


**Figure S1: Colony forming units (bacteria and fungi-like) inhabiting *P. xylostella*.** Ten larvae of *P. xylostella* were fed with the artificial diet used for the experiment without added bacteria and collected and extracted after 5 days and 7 days. The 5 days time-point corresponds to 2^nd^ instar larvae used at the start of the experiment for feeding with *P. protegens* CHA0. Extracts were plated onto LB media and incubated at 18 °C, 24 °C and 37 °C for one week. Boxplots correspond to 10 extracted larvae.

**
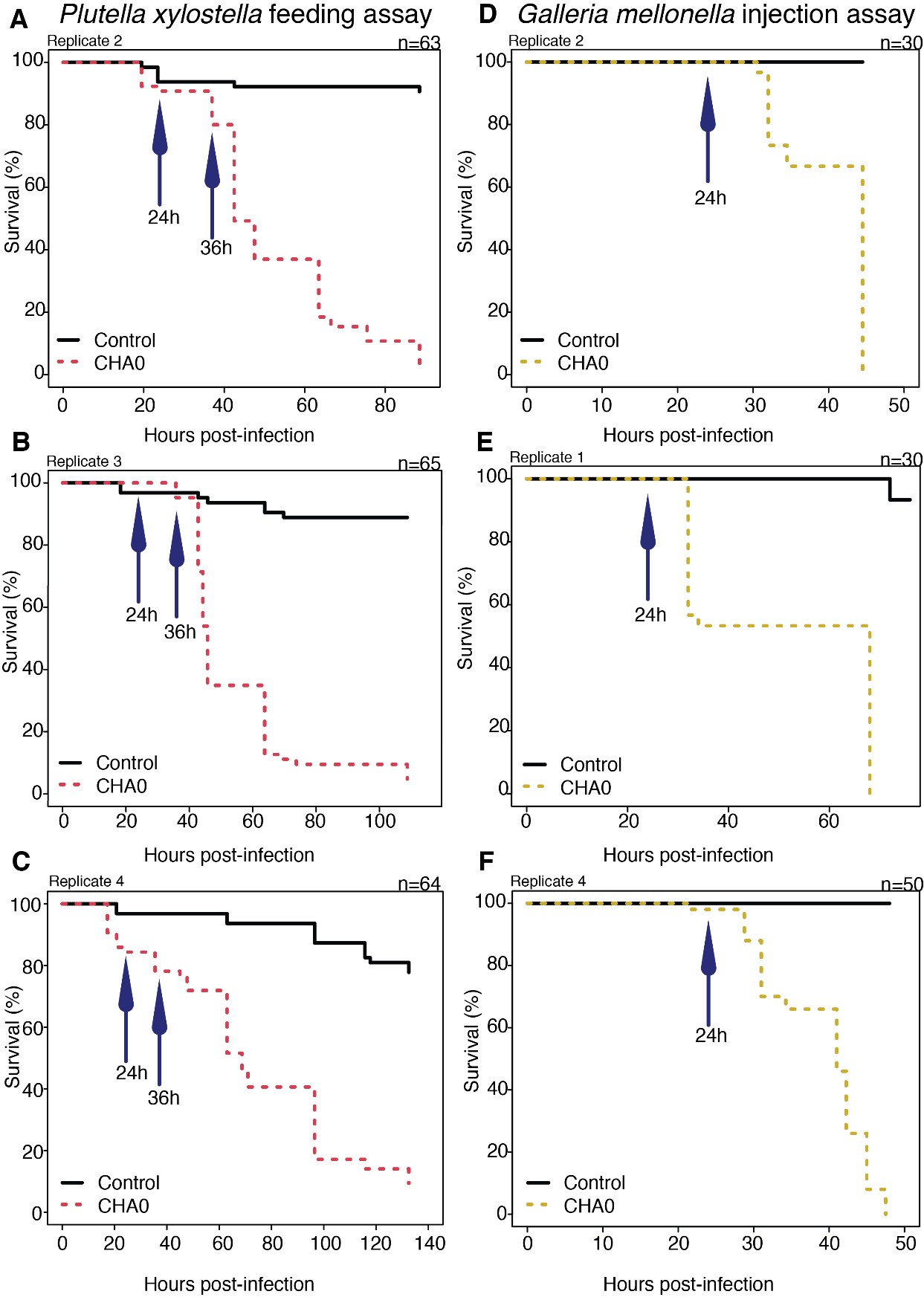
**

**Figure S2. Survival of *Plutella xylostella* (left panels) and *Galleria mellonella* (right panels) larvae after inoculation with *P. protegens* CHA0.** A-C) *P. xylostella* larvae were exposed to artificial diet pellets spiked with 4·x 10^6^ CHA0 cells. D-F) Seventh instar *G. mellonella* larvae were injected with 2·x 10^3^ CHA0 cells. At the time points indicated by arrows a part of the larvae were used for RNA extraction. The replicate number and the numbers of larvae used for calculation of the survival curves are indicated on top of the panels.


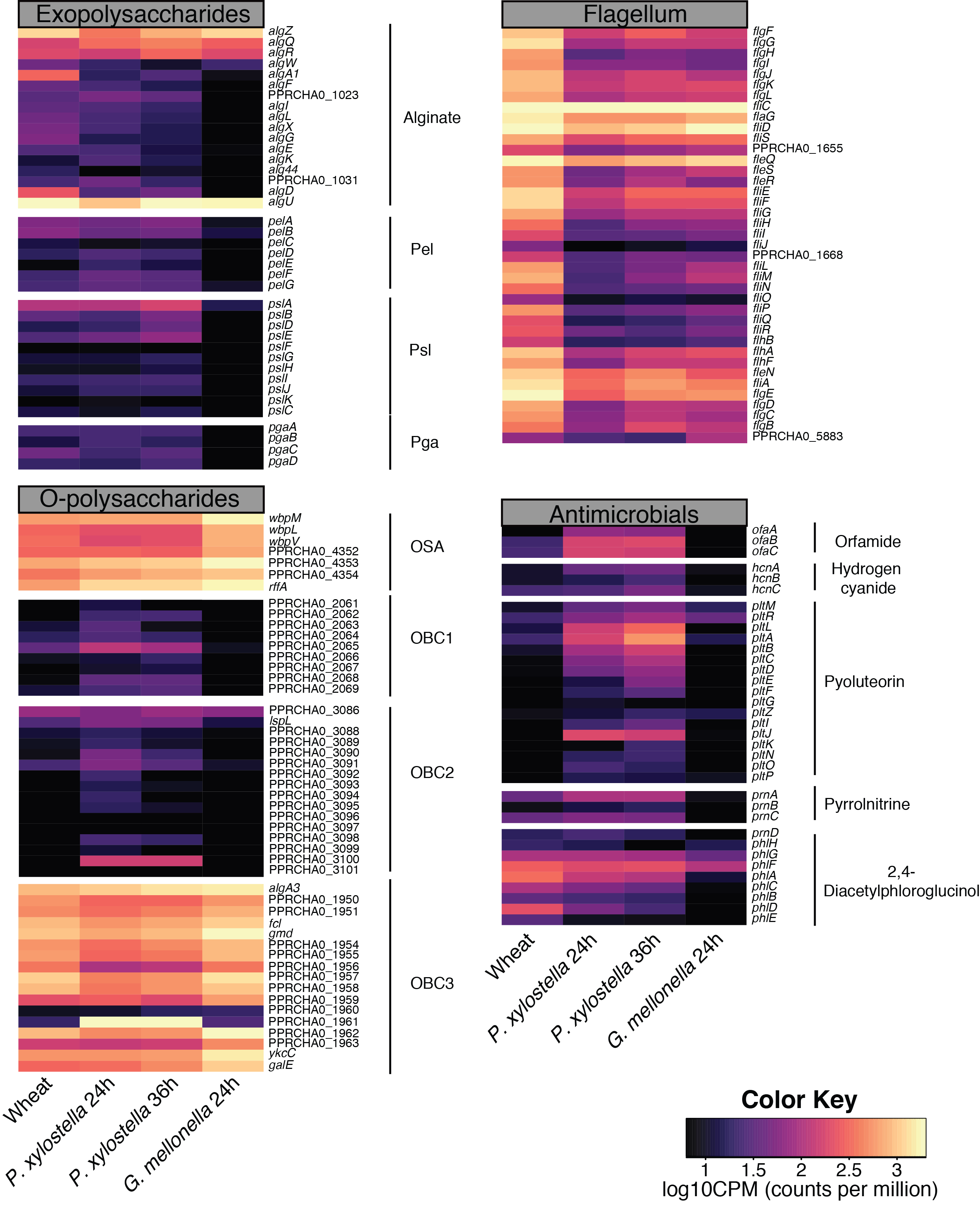


**Figure S3.** **Heatmap showing the normalized reads (counts per million) for genes related to exopolysaccharides, o-polyaccharides, flagellum and antimicrobials in *P. protegens* CHA0 colonizing different hosts.** Black indicates low expression (less than 10 counts per million reads) and yellow indicates high expression (more than 10^3^ counts per million reads). The “Wheat” sample corresponds to wheat-roots 1 week after inoculation, “*P. xylostella* 24h/36h” to *Plutella xylostella* 24 h and 36 h after oral infection; “*G. mellonella”* to *Galleria mellonella* hemolymph 24 h after hemocoel injection.

**
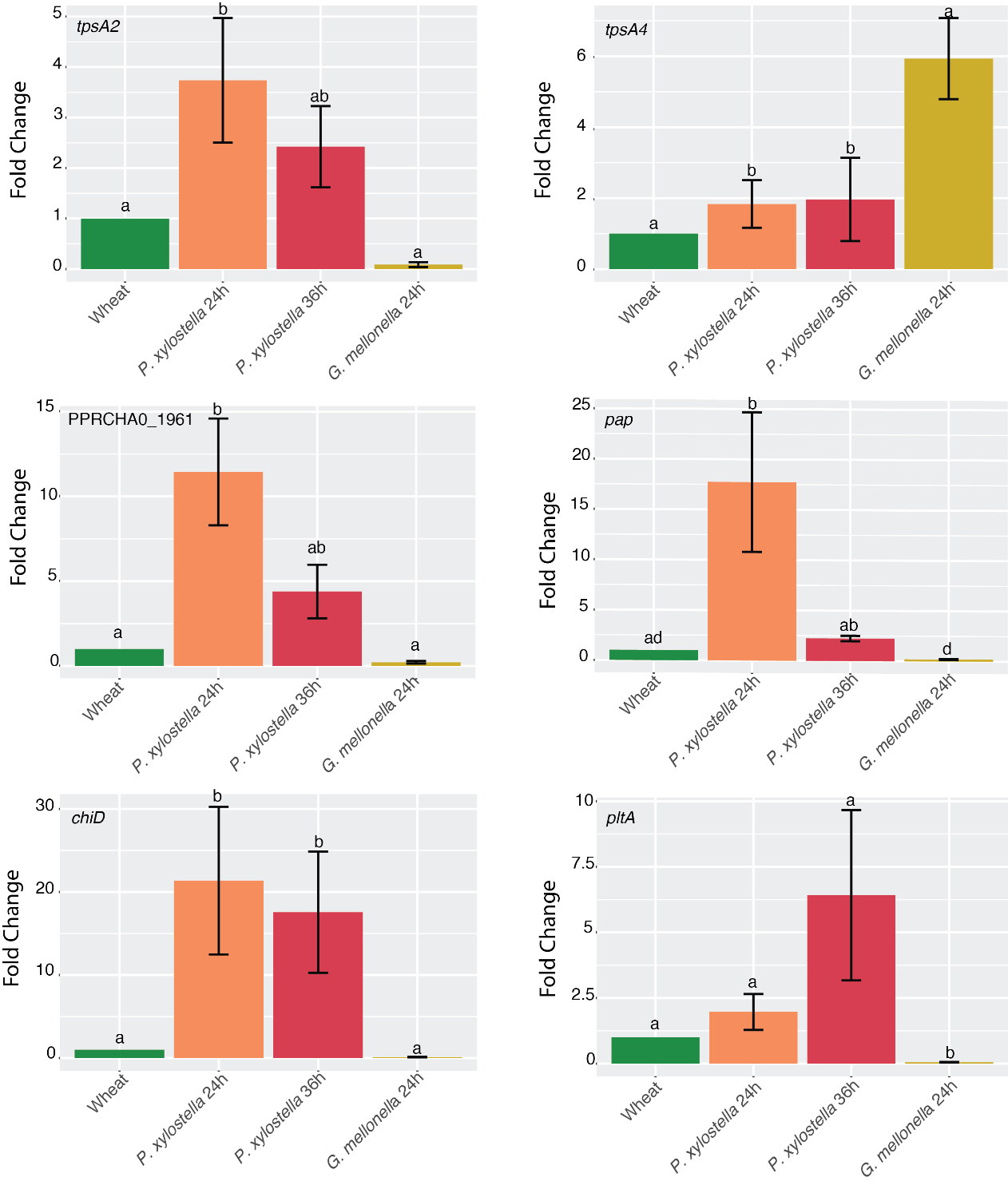
**

**Figure S4. Confirmation of RNA-sequencing results by qPCR.** Quantitative PCR was performed on *tpsA2*, *tpsA4*, transposase PPRCHA0_1961, *pap*, *chiD*, and *pltA* genes with the same samples as used for RNA-sequencing. The figure shows gene expression normalized to that on wheat roots (normalized gene expression on wheat roots = 1). Columns with different letters are statistically different at *P*<0.05 (Kruskal-Wallis analysis followed by Dunn’s post hoc test in R).


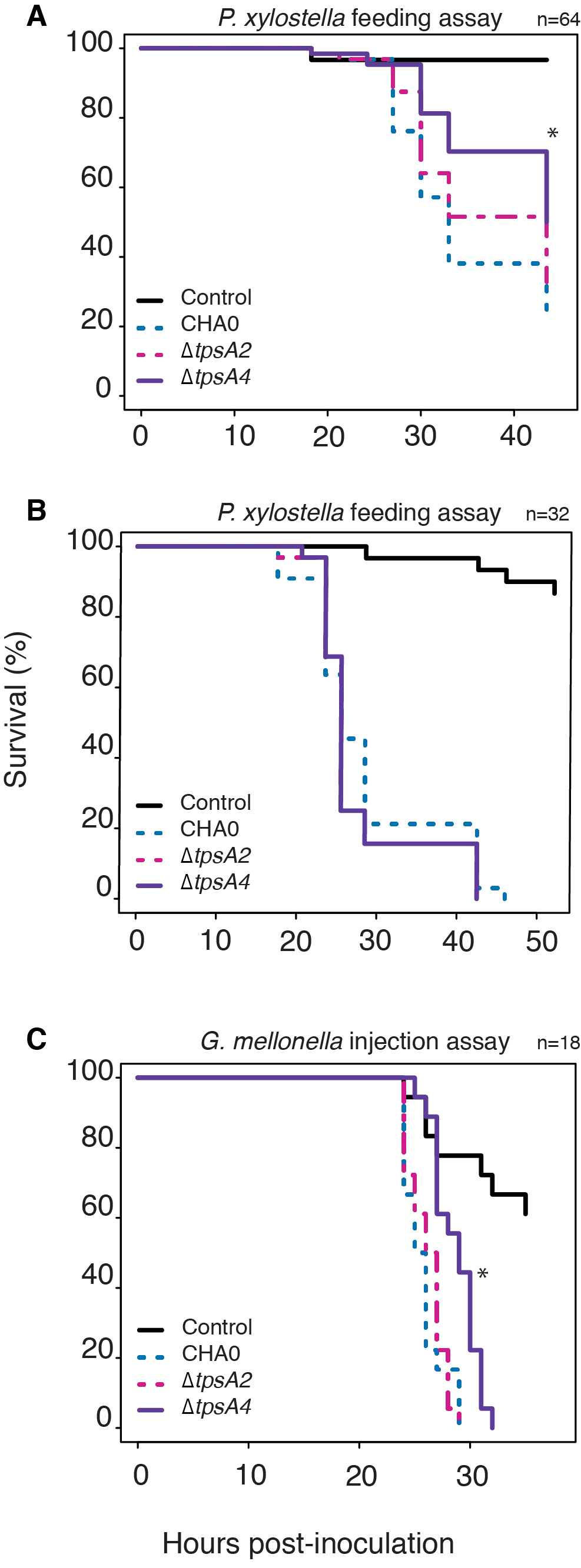


**Figure S5. Survival of *Plutella xylostella* and *Galleria mellonella* larvae when treated with Δ*tpsA2* and Δ*tpsA4* mutants of *P. protegens* CHA0.** A-B) Second instar *P. xylostella* larvae were exposed to artificial diet pellets spiked with 4·x 10^6^ cells of *tpsA2* or *tpsA4* deletion mutants. Thirty-two or sixty-four larvae were used per bacterial strain. C) Seventh instar *G. mellonella* larvae were injected with 2 x 10^3^ cells of *tpsA2* or *tpsA4* deletion mutants into the hemocoel. Eighteen larvae were used per bacterial strain. For all experiments, significant differences were assessed between bacterial treatments by log-rank test with a p-value<0.05.


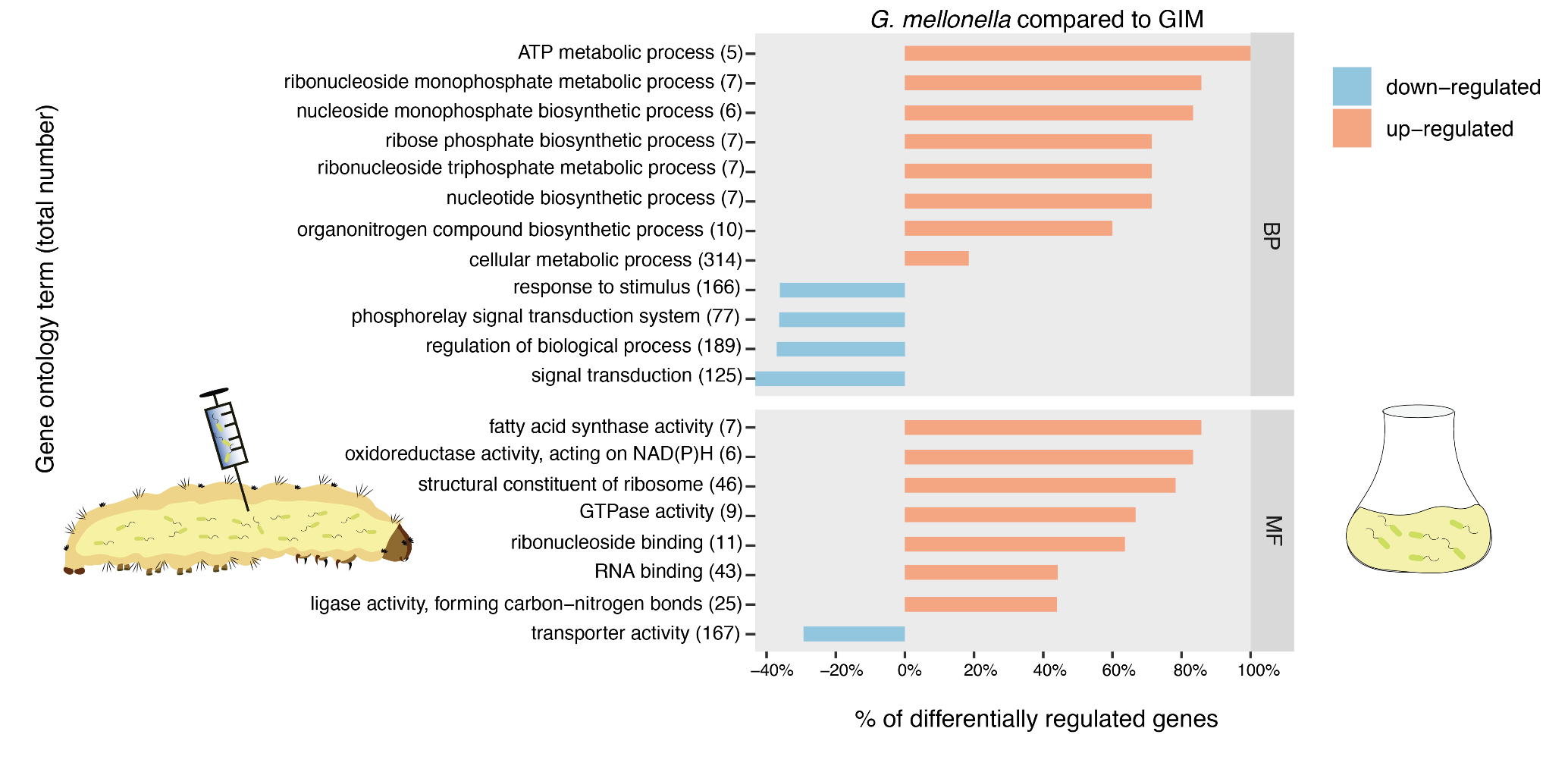


**Figure S6.** **Comparison of the transcriptomes of *P. protegens* CHA0 during colonization of the hemolymph of *Galleria mellonella* and Grace’s insect medium.** CHA0 transcriptomes were compared using the general linear model pipeline of edgeR package in R. *G. mellonella* was compared to GIM as a reference. Total differentially expressed genes were subjected to a GO enrichment analysis. Significant GO terms for the given set of genes are shown. Total genes related to a GO term present in the CHA0 genome are given between brackets and the indicated percentage shows how many of those are differentially expressed in the comparison (p-value<0.001). “*G. mellonella”* corresponds to*Galleria mellonella* hemolymph 24 h after hemocoel injection, and “GIM” to Grace Insect medium.

**
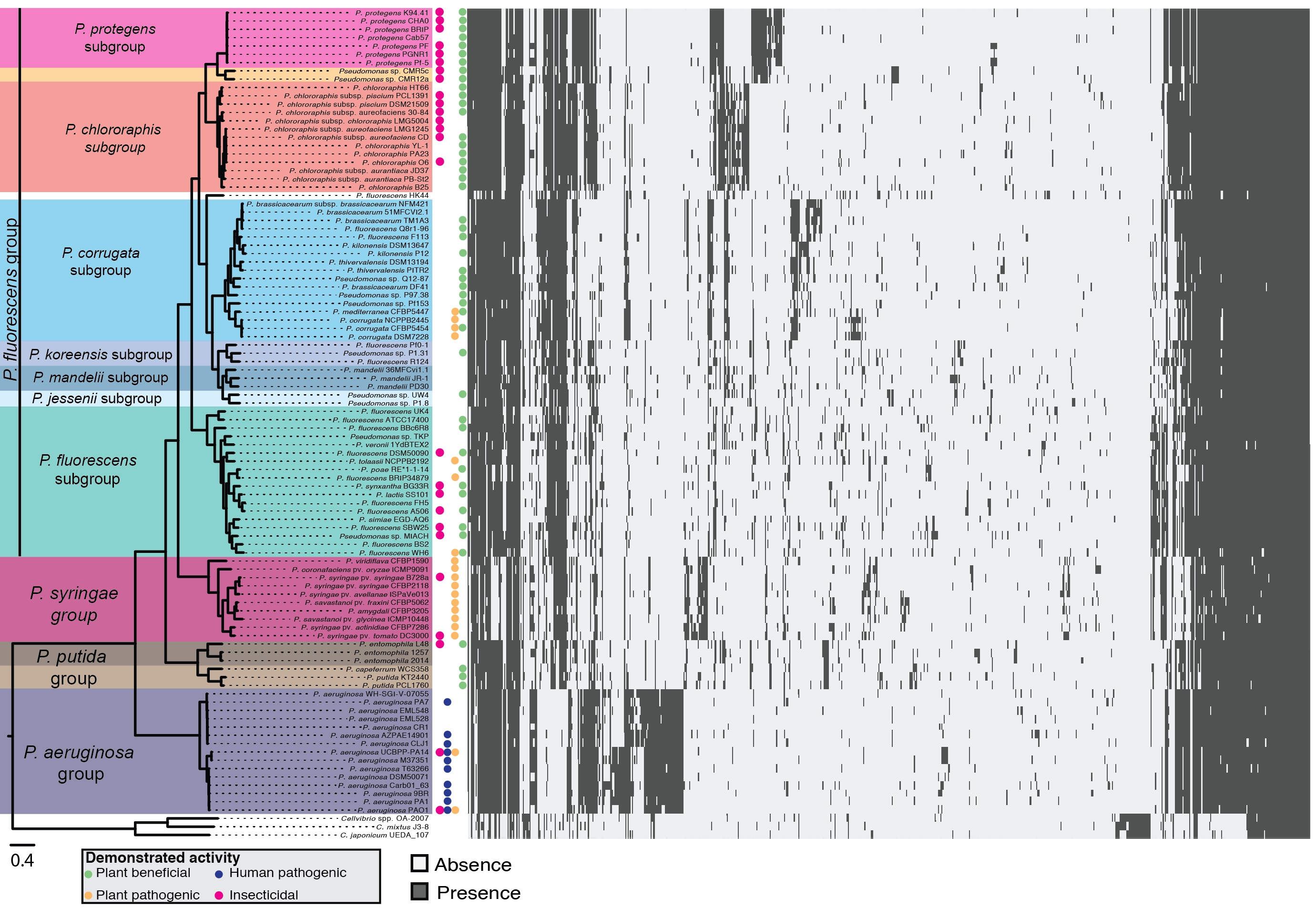
**

**Figure S7. Orthologue comparison based on whole proteomes between different strains of *Pseudomonas*.** Comparison of the full *in silico* proteomes of 97 pseudomonads belonging to phylogenetic groups harbouring insect pathogenic, human pathogenic, plant pathogenic and plant beneficial strains (groups and subgroups as defined by Hesse et al., [9]. Strain with described activity are marked in: pink for insecticidal activity (oral or injectable); dark-blue for human pathogenic activity; orange for plant pathogenic activity; green for plant-beneficial activity (references in Supplementary Table S2). Abbreviations: Am.: amidases; Enz.: enzymes.

**Supplementary Tables**

**Supplementary Table S1. Reads obtained from Illumina NextSeq sequencing of RNA extracted from different CHA0 inoculated host/environments.** RNA 260/280 = absorbance ratio for RNA quality. Total reads= reads after trimming and quality check. Mapped= reads mapped against the *P. protegens* CHA0 genome; Counted= reads mapped to conding sequences. Wheat = wheat roots 1 week after inoculation, *P. xylostella* 24h/36h = *Plutella xylostella* 24 h and 36 h after oral infection; *G. mellonella* = *Galleria mellonella* hemolymph 24 h after hemocoel injection; LB = lysogeny broth; GIM = Grace’s insect medium.

| Condition | Sample | RNA 260/280 | Reads | | | | |
| --- | --- | --- | --- | --- | --- | --- | --- |
|  |  |  | Total | Mapped | % Mapped | Counted | % Counted |
| Wheat | 1 | 2.11 | 75 992 549 | 4 390 592 | 5.78 | 3 682 414 | 4.85 |
| Wheat | 2 | 2.05 | 68 841 535 | 7 606 894 | 11.05 | 6 403 568 | 9.30 |
| Wheat | 3 | 2.13 | 42 211 861 | 3 081 907 | 7.30 | 2 692 569 | 6.38 |
| Wheat | 4 | 2.09 | 42 066 805 | 2 459 093 | 5.85 | 2 108 168 | 5.01 |
| *Plutella*24h | 1 | 2.14 | 225 647 887 | 191 077 | 0.08 | 96 263 | 0.04 |
| *Plutella*24h | 2 | 2.15 | 291 918 440 | 209 593 | 0.07 | 134 556 | 0.05 |
| *Plutella2*4h | 3 | 2.14 | 97 403 265 | 63 368 | 0.07 | 42 551 | 0.04 |
| *Plutella*24h | 4 | 2.16 | 120 197 312 | 142 690 | 0.12 | 91 670 | 0.08 |
| *Plutella*36h | 1 | 2.15 | 241 594 068 | 248 573 | 0.10 | 129 985 | 0.05 |
| *Plutella*36h | 2 | 2.14 | 21 2740 597 | 675 097 | 0.32 | 414 905 | 0.20 |
| *Plutella*36h | 3 | 2.16 | 127 029 836 | 500 219 | 0.39 | 264 853 | 0.21 |
| *Plutella*36h | 4 | 2.17 | 123 956 872 | 124 692 | 0.10 | 83 437 | 0.07 |
| *Galleria* | 1 | 2.15 | 135 529 889 | 21 861 431 | 16.13 | 10 635 551 | 7.85 |
| *Galleria* | 2 | 2.12 | 129 063 190 | 7 148 913 | 5.54 | 3 445 638 | 2.67 |
| *Galleria* | 3 | 2.1 | 48 399 955 | 8 630 776 | 17.83 | 3 918 036 | 8.10 |
| *Galleria* | 4 | 2.17 | 48 333 173 | 19 985 280 | 41.35 | 10 563 308 | 21.86 |
| LB | 1 | 2.13 | 61 202 272 | 60 356 041 | 98.62 | 27 122 810 | 44.32 |
| LB | 2 | 2.13 | 66 428 498 | 65 953 001 | 99.28 | 31 134 777 | 46.87 |
| LB | 3 | 2.13 | 63 401 498 | 62 486 186 | 98.56 | 35 990 967 | 56.77 |
| LB | 4 | 2.13 | 88 252 664 | 87 093 887 | 98.69 | 48 536 989 | 55.00 |
| GIM | 1 | 2.13 | 56 654 217 | 55 975 156 | 98.80 | 23 073 397 | 40.73 |
| GIM | 2 | 2.08 | 65 782 951 | 65 419 039 | 99.45 | 21 209 037 | 32.24 |
| GIM | 3 | 2.12 | 80 495 439 | 79 761 444 | 99.09 | 30 392 689 | 37.76 |
| GIM | 4 | 2.13 | 79 355 862 | 78 784 965 | 99.28 | 29 412 337 | 37.06 |

**Table S2**. **List of *Pseudomonas* strains used in the orthologue analysis. IP = Insect pathogen. PP = Plant Pathogen. HP = Human Pathogen. PB =Plant Beneficial.**

| **Strain** | **Isolation** | **Activity** | **References** | **Assembly** |
| --- | --- | --- | --- | --- |
| *P. protegens* K94.41 | Cucumber root | IP, PB | [10] | GCF_001269485.1_ASM126948v1 |
| *P. protegens* CHA0 | Tobacco root | IP, PB | [11] | GCF_900560965.1_PPRCHA0 |
| *P. protegens* BRIP | Cyclops | IP, PB | [12] | GCF_001269495.1_ASM126949v1 |
| *P. protegens* Cab57 | Shepherd's purse | PB | [13] | GCF_000828695.1_ASM82869v1 |
| *P. protegens* PF | Wheat leaf | IP, PB | [14] | GCF_001269465.1_ASM126946v1 |
| *P. protegens* PGNR1 | Tobacco root | IP, PB | [15] | GCF_001269475.1_ASM126947v1 |
| *P. protegens* Pf-5 | soil | IP, PB | [16] | GCF_000012265.1_ASM1226v1 |
| *Pseudomonas* sp. CMR5c | Red cocoyan | IP, PB | [17, 18] | GCF_003850545.1_ASM385054v1 |
| *Pseudomonas* sp. CMR12a | Red cocoyan | IP, PB | [17, 18] | GCF_003850565.1_ASM385056v1 |
| *P. chlororaphis* HT66 | Rice root | PB | [19] | GCF_000597925.1_ASM59792v1 |
| *P. chlororaphis* subsp. piscium PCL1391 | Tomato root | IP, PB | [20] | GCF_003850445.1_ASM385044v1 |
| *P. chlororaphis* subsp. piscium DSM 21509 | European perch | IP, PB | [21] | GCF_003850345.1_ASM385034v1 |
| *P. chlororaphis* subsp. aureofaciens 30-84 | wheat seed | IP, PB | [22] | GCF_000281915.1_ASM28191v1 |
| *P. chlororaphis* subsp. chlororaphis LMG 5004 | Contaminated plate | IP | [23] | GCF_001269625.1_ASM126962v1 |
| *P. chlororaphis* subsp. aureofaciens LMG 1245 | River Clay | IP | [24] | GCF_001269575.1_ASM126957v1 |
| *P. chlororaphis* subsp. aureofaciens CD | Cyclops | IP, PB | [12, 17] | GCF_001269595.1_ASM126959v1 |
| *P. chlororaphis* YL-1 | Soybean root tip | PB | [25] | GCF_000512485.1_PCYL_1 |
| *P. chlororaphis* PA23 | Soybean | PB | [26, 27] | GCF_000698865.1_ASM69886v1 |
| *P. chlororaphis* O6 | Wheat root | IP, PB | [28] | GCF_000264555.1_ASM26455v1 |
| *P. chlororaphis* subsp. aurantiaca JD37 | Other | PB | [29] | GCF_000761195.1_ASM76119v1 |
| *P. chlororaphis* subsp. aurantiaca PB-St2 | Sugar cane | PB | [30] | GCF_000506385.1_PcsubspaPBSt2-2.0 |
| *P. chlororaphis* B25 | Plant | PB | [31] | GCF_003851985.1_ASM385198v1 |
| *P. fluorescens* HK44 | ingeenered |  | [32] | GCF_000217955.2_PfluHK442.0 |
| *P. brassicacearum* subsp. brassicacearum NFM421 | Arabidopsis |  | [33] | GCF_000194805.1_ASM19480v1 |
| *P. brassicacearum* 51MFCVI2.1 | Arabidopsis |  | [34] | GCF_000510785.1_ASM51078v1 |
| *P. brassicacearum* TM1A3 | Tomato root | PB | [35] | GCF_001269635.1_ASM126963v1 |
| *P. fluorescens* Q8r1-96 | soil | PB | [36] | GCF_000263695.1_ASM26369v2 |
| *P. fluorescens* F113 | Sugar beet root | PB | [37] | GCF_000237065.1_ASM23706v1 |
| *P. kilonensis* DSM 13647 | soil |  | [17, 38] | GCF_001269885.1_ASM126988v1 |
| *P. kilonensis* P12 | Tobacco root | PB | [17, 35] | GCF_001269725.1_ASM126972v1 |
| *P. thivervalensis* DSM 13194 | Rapeseed |  | [17, 33] | GCF_001269655.1_ASM126965v1 |
| *P. thivervalensis* PITR2 | Wheat root | PB | [17, 35] | GCF_001269685.1_ASM126968v1 |
| *Pseudomonas* sp. Q12-87 | Wheat root | PB | [17, 35] | GCF_001269755.1_ASM126975v1 |
| *P. brassicacearum* DF41 | Canola | PB | [27] | GCF_000585995.1_ASM58599v1 |
| *Pseudomonas* sp. P97.38 | Cucumber root | PB | [17, 10] | GCF_001269745.1_ASM126974v1 |
| *Pseudomonas* sp. Pf153 | Cucumber root | PB | [17, 39] | GCF_001269775.1_ASM126977v1 |
| *P. mediterranea* CFBP 5447 | Tomato stem | PP, PB | [40, 41] | GCF_000774145.1_ASM77414v1 |
| *P. corrugata* NCPPB2445 | Tomato stem | PP | [42, 43] | GCF_001411965.1_ASM141196v1 |
| *P. corrugata* CFBP 5454 | Tomato stem | PP, PB | [44, 45] | GCF_000522485.1_Pco1 |
| *P. corrugata* DSM 7228 | Tomato stem | PP | [17, 43] | GCF_001269905.1_ASM126990v1 |
| *P. fluorescens* Pf0-1 | Soil |  | [46–48] | GCF_000012445.1_ASM1244v1 |
| *Pseudomonas* sp. P1.31 | Woodlouse | PB | [12, 17] | GCF_001269815.1_ASM126981v1 |
| *P. fluorescens* R124 | tepui |  | [49] | GCF_000292795.1_PF-R124.01 |
| *P. mandelii* 36MFCvi1.1 |  |  |  | GCF_000381285.1_ASM38128v1 |
| *P. mandelii* JR-1 | Water |  | [50, 51] | GCF_000257545.3_ASM25754v3 |
| *P. mandelii* PD30 | soil |  | [52, 53] | GCF_000690555.2_Pmandelii1.0 |
| *Pseudomonas* sp. UW4 | Reed root | PB | [54] | GCF_000316175.1_ASM31617v1 |
| *Pseudomonas* sp. P1.8 | Earthworm |  | [12, 17] | GCF_001269805.1_ASM126980v1 |
| *P. fluorescens* UK4 | Water |  | [55, 56] | GCF_000730425.1_ASM73042v1 |
| *P. fluorescens* ATCC 17400 | Hen egg | PB | [57] | GCF_000708695.2_ATCC_17400 |
| *P. fluorescens* BBc6R8 | fungus | PB | [58, 59] | GCF_000297195.2_Pseudomonas.strain_BBc6R8_v2.0 |
| *Pseudomonas* sp. TKP | HCH soil |  | [60] | GCF_000508205.1_ASM50820v1 |
| *P. veronii* 1YdBTEX2 | Contaminated soil |  | [61] | GCF_000350565.1_P.ver1YdBTEX2v.1 |
| *P. fluorescens* DSM 50090 | Prefilter tanks | IP, PB | [17, 62] | GCF_001269845.1_ASM126984v1 |
| *P. tolaasii* NCPPB 2192 | Mushrom | PP | [63, 64] | GCF_002813445.1_ASM281344v1 |
| *P. poae* RE*1.1.14 | Sugar Beet root | PB | [65, 66] | GCF_000336465.1_ASM33646v1 |
| *P. fluorescens* BRIP34879 | Barley glume | PP | [67] | GCF_000334015.1_BRIP34879v2.0 |
| *P. synxantha* BG33R | Peach tree root | IP, PB | [68, 69] | GCF_000263715.2_ASM26371v2 |
| *P. lactis* SS101 | Wheat root | IP, PB | [17, 70] | GCF_000263675.1_ASM26367v2 |
| *P. fluorescens* FH5 | Fresh water |  | [71] | GCF_000511155.2_v1 |
| *P. fluorescens* A506 | Pear tree leaf | IP, PB | [69, 72, 73] | GCF_000262325.2_ASM26232v2 |
| *P. simiae* EGD-AQ6 | Sewage Sludge |  | [74] | GCF_000465595.1_GS_De_Novo_Assembly |
| *P. fluorescens* SBW25 | Sugar beet leaves | IP, PB | [47, 75] | GCF_000009225.2_ASM922v1 |
| *Pseudomonas* sp. MIACH | Wheat root | IP, PB | [17, 76] | GCF_001269925.1_ASM126992v1 |
| *P. fluorescens* BS2 | Soil |  | [77] | GCF_000308175.1_PseuFluoBS2 |
| *P. fluorescens* WH6 | Wheat root | PP, PB | [78, 79] | GCF_000166515.1_WH6_v1 |
| *P. viridiflava* CFBP 1590 | Diseased Cherry | PP | [80] | GCF_900184295.1_Chr_1 |
| *P. coronafaciens* pv. oryzae ICMP 9091 | rice leaf | PP | [81] | GCF_003701785.1_ASM370178v1 |
| *P. syringae* pv. syringae B728a | Bean leaf | IP, PP | [82–84] | GCF_000012245.1_ASM1224v1 |
| *P. syringae* pv. syringae CFBP2118 | Sweet Cherry | PP | [85] | GCF_900235865.1_CFBP2118 |
| *P. syringae* pv. avellanae str. ISPaVe013 | hazelnut | PP | [86] | GCF_000302795.1_Pav013_1.0 |
| *P. savastanoi* pv. fraxini CFBP 5062 | Ash | PP | [87] | GCF_001538155.1_ASM153815v1 |
| *P. amygdali* CFBP 3205 | Almond tree leaf | PP | [88, 89] | GCF_000935645.1_PSAVPseNe107-G1 |
| *P. savastanoi* pv. glycinea ICMP 10448 | Soybean | PP | [90] | GCF_003699735.1_ASM369973v1 |
| *P. syringae* pv. actinidiae CFBP 7286 | Kiwi fruit | PP | [91, 92] | GCF_000245415.1_ASM24541v1 |
| *P. syringae* pv. tomato str. DC3000 | Tomato | IP, PP | [82, 93, 94] | GCF_000007805.1_ASM780v1 |
| *P. entomophila* L48 | Drosophila | IP, PB | [95, 96] | GCF_000026105.1_ASM2610v1 |
| *P. entomophila* 1257 | Soil |  |  | GCF_003940825.1_ASM394082v1 |
| *P. entomophila* 2014 | Soil |  | | GCF_003940785.1_ASM394078v1 |
| *P. capeferrum* WCS358 | Potato root | PB | [64, 97, 98] | GCF_000731675.1_ASM73167v1 |
| *P. putida* KT2440 | Soil | PB | [96, 99–101] | GCF_000007565.2_ASM756v2 |
| *P. putida* PCL1760 | Avocado root | PB | [102] | GCF_001282125.1_ASM128212v1 |
| *P. aeruginosa* WH-SGI-V-07055 | Clinical isolate |  | [103, 104] | GCF_001450355.1_WH-SGI-V-07055 |
| *P. aeruginosa* PA7 | non-respiratory human isolate | HP | [104–107] | GCF_000017205.1_ASM1720v1 |
| *P. aeruginosa* EML548 | unknown |  | [104, 107, 108] | GCF_001280765.1_ASM128076v1 |
| *P. aeruginosa* EML528 | unknown |  | [104, 107, 109] | GCF_001280755.1_ASM128075v1 |
| *P. aeruginosa* CR1 | Chili root |  | [104] | GCF_003025345.2_ASM302534v2 |
| *P. aeruginosa* AZPAE14901 | Pus | HP | [110] | GCF_000791105.1_AZPAE14901 |
| *P. aeruginosa* CLJ1 | Hemorrhagic pneumonia | HP | [105, 106] | GCF_003032395.1_ASM303239v1 |
| *P. aeruginosa* UCBPP-PA14 | Human burn | IP, PP, HP | [104, 111–114] | GCF_000014625.1_ASM1462v1 |
| *P. aeruginosa* M37351 | Clinical isolate | HP | [104] | GCF_001516385.1_ASM151638v1 |
| *P. aeruginosa* T63266 | Clinical isolate | HP | [104] | GCF_001516105.1_ASM151610v1 |
| *P. aeruginosa* DSM 50071 | unknown |  | [115] | GCF_001042925.1_G1273 |
| *P. aeruginosa* Carb01 63 | Hospital sink | HP | [116] | GCF_000981825.1_ASM98182v1 |
| *P. aeruginosa* 9BR | Cystic Fibrosis patient | HP | [104, 117] | GCF_000223925.1_ASM22392v2 |
| *P. aeruginosa* PA1 | Lung infection | HP | [104, 118] | GCF_000496605.2_ASM49660v2 |
| *P. aeruginosa* PAO1 | Wound | IP, PP, HP | [99, 106, 114, 119] | GCF_000006765.1_ASM676v1 |
| *Cellvibrio* spp. OA.2007 | Activated sludge |  | [120] | GCF_000953825.1_ASM95382v1 |
| *C. mixtus* J3.8 | Giant snail |  | [121] | GCF_002268635.1_ASM226863v1 |
| *C. japonicum* UEDA 107 | Soil |  | [122] | GCF_000019225.1_ASM1922v1 |

**Supplementary Table S3. Primers used for RT-qPCR.**

| **Gene** | **Sequence** | **Annealing T°C** | **bp** | **Reference** |
| --- | --- | --- | --- | --- |
| *chiD* | ATCATCCGTCTGGTGGAAACC |  |  |  |
|  | TGATGATGAAGTGCTTGCCCT | 60 °C | 154 | This study |
| *tpsA1* | TGATCCTCAACAACGCCATCA |  |  |  |
|  | ATCTGACCGTTCTCAACCACC | 65 °C | 277 | This study |
| *tpsA2* | TACGCCAAGAAGCTCAACGT |  |  |  |
|  | CGTTGGCGTCGATCTGGATA | 65 °C | 235 | This study |
| *tpsA4* | GTCAACATCGTCGCGCCCAA |  |  |  |
|  | TGAGTTGCGAGGCATTGCGG | 65 °C | 208 | This study |
| *pap* | AAGAAACCTACGATGCCGAGG |  |  |  |
|  | CATTCGTTGAGCAGCTTGACC | 60 °C | 154 | This study |
| *pltA* | CGATTCACTCCTGGTTCGACA |  |  |  |
|  | TCGGAGTTGGTGTAGTTCTGC | 60 °C | 181 | This study |
| PPRCHA0_1961 | GTACGCCTTTATCAAGCTGCG |  |  |  |
|  | TAACCGCTGGGATGGACTTTC | 62 °C | 81 | This study |
| rRNA 16S | ACTTTAAGTTGGGAGGAAGGG |  |  |  |
|  | ACACAGGAAATTCCACCACCC | 60 °C | 251 | [123] |

**Supplementary Table S4. Quantitative PCR conditions for LightCycler480 (Roche, Switzerland)**

|  | **Phase** | **°C** | **Acquisition mode** | **Time** | **Ramp Rate (°C/s)** | **Acquisitions (per °C)** |
| --- | --- | --- | --- | --- | --- | --- |
|  | Preincubation | 95 |  | 15 min |  |  |
| Quant. | Amplification | 95 | none | 15 s | 4.4 |  |
|  |  | * | none | 20 s | 2.2 |  |
|  |  | 72 | Single/none** | 30 s | 4.4 |  |
|  |  | ** | none | 5 s | 4.8 |  |
| Melting | Melting Curve | 95 | none | 5 s | 4.4 |  |
|  |  | 65 | none | 1 min | 2.2 |  |
|  |  | 97 | continuous | - | 0.06 | 10 |
|  | Cooling | 40 | none | 30 s | 2.2 |  |

*Different temperatures from Supplementary Table S1 for the corresponding primers.

** To avoid false amplification due to primer dimer, amplification protocols had an extra step at 86°C for *tpsA2* and at 83°C for PPRCHA0_1961 and *pap*. In absence of primer dimers, this step was not included.

**Table S5. Plasmids and primers used to create *tpsA2* and *tpsA4* deletion mutants**

| **Plasmid or primer** | **Relevant characteristics or sequence** | **Reference** |
| --- | --- | --- |
| Plasmids |  |  |
| pEMG | pSEVA212S; oriR6K, lacZα MCS flanked by two I-SceI sites; Kmr, Apr | [7] |
| pSW-2 | oriRK2, xylS, Pm::I-sceI; Gmr | [7] |
| Primers |  |  |
| *tpsA2*-1 | 5’-CGGAATTCACCGCATCACCGAAAGCCAGCT-3’ - EcoRI | This study |
| *tpsA2*-2 | 5’-GGGGTACCTACGTCCATGTGCGAATCATCC-3’ - KpnI | This study |
| *tpsA2*-3 | 5’-GGGGTACCGATGCCAATGGCAAGGACACTA-3’ - KpnI | This study |
| *tpsA2*-4 | 5’-CGGGATCCCAGCACCAATACCTGACCTCAT-3’ - BamHI | This study |
| *tpsA4*-1 | 5’-CGGAATTCACTCGCAGCAGGTACAGCGCAA-3’ - EcoRI | This study |
| *tpsA4*-2 | 5’-GGGGTACCGCCCTGGGGTGAAAGGTTGAAT-3’ - KpnI | This study |
| *tpsA4*-3 | 5’-GGGGTACCCTGTCCAAGTCACCGGTCAACC-3’ – KpnI | This study |
| *tpsA4*-4 | 5’-CGGGATCCGTCCAACTTGGGAATGCAGATA-3 - BamHI | This study |

**Supplementary Table S7. Similarities of the predicted two-partner secretion A (TpsA) – like proteins of *P. protegens* CHA0 with related proteins in pathogenic bacteria.**

|  | **Protein in *P. protegens* CHA0 (gen ID)** | | | | |
| --- | --- | --- | --- | --- | --- |
| **Organism** | **Protein Homologue (gen ID)** | **TpsA1** (PPRCHA0_0169) | **TpsA2** (PPRCHA0_0626) | **TpsA3** (PPRCHA0_1575) | **TpsA4** (PPRCHA0_4278) |
| *Pseudomonas aeruginosa* PA7 | ExlA (PSPA7_4642) | 44.35% | 39.35% | 43.85% | 59.53% |
| *Serratia. marcescens* | ShlA (A8A12_12190) | 33.69% | 28.82% | 31.85% | 35.34% |
| *Proteus mirabilis* | HmpA (F4W58_04730) | 32.46% | 31.58% | 29.66% | 31.22% |
| *Photorhabdus. luminescens* | PhlA (TP56_RS21180) | 33.64% | 31.75% | 29.07% | 32.19% |
| *Bordetella pertussis* | FhaB (L565_RS14220) | 38.27% | 28.04% | 37.23% | 37.23% |
| *Haemophilus. influenza* | HMW1A  (ADC26_RS06775) | 29.21% | 27.42% | 29.31% | 26.29% |

**References**

1. King EO, Ward MK, Raney DE. Two simple media for the demonstration of pyocyanin and fluorescin. *J Lab Clin Med* 1954; **44**: 301–307.

2. Landa BB, de Werd HAE, McSpadden Gardener BB, Weller DM. Comparison of three methods for monitoring populations of different genotypes of 2,4-diacetylphloroglucinol-producing *Pseudomonas fluorescens* in the rhizosphere. *Phytopathology* 2002; **92**: 129–137.

3. Flury P, Vesga P, Péchy-Tarr M, Aellen N, Dennert F, Hofer N, et al. Antimicrobial and insecticidal: cyclic lipopeptides and hydrogen cyanide produced by plant-beneficial *Pseudomonas* strains CHA0, CMR12a, and PCL1391 contribute to insect killing. *Front Microbiol* 2017; **8**.

4. Flury P, Vesga P, Dominguez-Ferreras A, Tinguely C, Ullrich CI, Kleespies RG, et al. Persistence of root-colonizing *Pseudomonas protegens* in herbivorous insects throughout different developmental stages and dispersal to new host plants. *ISME J* 2019; **13**: 860–872.

5. Ruijter JM, Ramakers C, Hoogaars WMH, Karlen Y, Bakker O, van den Hoff MJB, et al. Amplification efficiency: linking baseline and bias in the analysis of quantitative PCR data. *Nucleic Acids Res* 2009; **37**: e45–e45.

6. Pfaffl MW. A new mathematical model for relative quantification in real-time RT-PCR. *Nucleic Acids Res* 2001; **29**: 45e–445.

7. Martínez-García E, de Lorenzo V. Engineering multiple genomic deletions in Gram-negative bacteria: analysis of the multi-resistant antibiotic profile of *Pseudomonas putida* KT2440. *Environ Microbiol* 2011; **13**: 2702–2716.

8. Kupferschmied P, Péchy-Tarr M, Imperiali N, Maurhofer M, Keel C. Domain shuffling in a sensor protein contributed to the evolution of insect pathogenicity in plant-beneficial *Pseudomonas protegens*. *PLoS Pathog* 2014; **10**: e1003964.

9. Hesse C, Schulz F, Bull CT, Shaffer BT, Yan Q, Shapiro N, et al. Genome-based evolutionary history of *Pseudomonas* spp. *Environ Microbiol* 2018; **20**: 2142–2159.

10. Wang C, Ramette A, Punjasamarnwong P, Zala M, Natsch A, Moënne-Loccoz Y, et al. Cosmopolitan distribution of phlD-containing dicotyledonous crop-associated biocontrol pseudomonads of worldwide origin. *FEMS Microbiol Ecol* 2001; **37**: 105–116.

11. Stutz EW, Défago G, Kern H. Naturally occurring fluorescent pseudomonads involved in suppression of black root rot of tobacco. *Phytopathology* 1986; **76**: 181–185.

12. Ruffner B, Péchy-Tarr M, Höfte M, Bloemberg G, Grunder J, Keel C, et al. Evolutionary patchwork of an insecticidal toxin shared between plant-associated pseudomonads and the insect pathogens *Photorhabdu*s and *Xenorhabdus*. *BMC Genomics* 2015; **16**: 609–623.

13. Takeuchi K, Noda N, Someya N. Complete genome sequence of the biocontrol strain *Pseudomonas protegens* Cab57 discovered in Japan reveals strain-specific diversity of this species. *PLoS ONE* 2014; **9**: e93683.

14. Levy E, Gough FJ, Berlin KD, Guiana PW, Smith JT. Inhibition of *Septoria tritici* and other phytopathogenic fungi and bacteria by *Pseudomonas fluorescens* and its antibiotics. *Plant Pathol* 1992; **41**: 335–341.

15. Keel C. A look into the toolbox of multi-talents: insect pathogenicity determinants of plant-beneficial pseudomonads. *Environ Microbiol* 2016; **18**: 3207–3209.

16. Loper JE, Hassan KA, Mavrodi DV, Davis EW, Lim CK, Shaffer BT, et al. Comparative genomics of plant-associated *Pseudomonas* spp.: insights into diversity and inheritance of traits involved in multitrophic interactions. *PLoS Genet* 2012; **8**.

17. Flury P, Aellen N, Ruffner B, Péchy-Tarr M, Fataar S, Metla Z, et al. Insect pathogenicity in plant-beneficial pseudomonads: phylogenetic distribution and comparative genomics. *ISME J* 2016; **10**: 2527–2542.

18. Perneel M, Heyrman J, Adiobo A, Maeyer KD, Raaijmakers JM, Vos PD, et al. Characterization of CMR5c and CMR12a, novel fluorescent *Pseudomonas* strains from the cocoyam rhizosphere with biocontrol activity. *J Appl Microbiol* 2007; **103**: 1007–1020.

19. Peng H, Zhang P, Bilal M, Wang W, Hu H, Zhang X. Enhanced biosynthesis of phenazine-1-carboxamide by engineered *Pseudomonas chlororaphis* HT66. *Microb Cell Factories* 2018; **17**.

20. Chin-A-Woeng TFC, Bloemberg GV, van der Bij AJ, van der Drift KMGM, Schripsema J, Kroon B, et al. Biocontrol by phenazine-1-carboxamide-producing *Pseudomonas chlororaphis* PCL1391 of tomato root rot caused by *Fusarium oxysporum* f. sp. radicis-lycopersici. *Mol Plant Microbe Interact* 1998; **11**: 1069–1077.

21. Burr SE, Gobeli S, Kuhnert P, Goldschmidt-Clermont E, Frey J. *Pseudomonas chlororaphis* subsp. piscium subsp. nov., isolated from freshwater fish. *Int J Syst Evol Microbiol* 2010; **60**: 2753–2757.

22. Thomashow LS, Weller DM, Bonsall RF, Pierson LS. Production of the antibiotic phenazine-1-Carboxylic Acid by fluorescent *Pseudomonas* species in the rhizosphere of wheat. *Appl Environ Microbiol* 1990; **56**: 908–912.

23. Peix A, Ramírez-Bahena M-H, Velázquez E. Historical evolution and current status of the taxonomy of genus *Pseudomonas*. *Infect Genet Evol* 2009; **9**: 1132–1147.

24. Kluyver AJ. *Pseudomonas aureofaciens* nov. spec. and its pigments. *J Bacteriol* 1956; **72**: 406–411.

25. Liu Y, Lu S-E, Baird SM, Qiao J, Du Y. Draft genome sequence of *Pseudomonas chlororaphis* YL-1, a biocontrol strain suppressing plant microbial pathogens. *Genome Announc* 2014; **2**: e01225-13.

26. Nandi M, Selin C, Brassinga AKC, Belmonte MF, Fernando WGD, Loewen PC, et al. Pyrrolnitrin and hydrogen cyanide production by *Pseudomonas chlororaphis* strain PA23 exhibits nematicidal and repellent activity against *Caenorhabditis elegans*. *PLoS ONE* 2015; **10**: e0123184.

27. Savchuk S, Dilantha Fernando WG. Effect of timing of application and population dynamics on the degree of biological control of *Sclerotinia sclerotiorum* by bacterial antagonists. *FEMS Microbiol Ecol* 2004; **49**: 379–388.

28. Park JY, Oh SA, Anderson AJ, Neiswender J, Kim J-C, Kim YC. Production of the antifungal compounds phenazine and pyrrolnitrin from *Pseudomonas chlororaphis* O6 is differentially regulated by glucose. *Lett Appl Microbiol* 2011; **52**: 532–537.

29. Fang R, Lin J, Yao S, Wang Y, Wang J, Zhou C, et al. Promotion of plant growth, biological control and induced systemic resistance in maize by *Pseudomonas aurantiaca* JD37. *Ann Microbiol* 2013; **63**: 1177–1185.

30. Mehnaz S, Bauer JS, Gross H. Complete genome sequence of the sugar cane endophyte *Pseudomonas aurantiaca* PB-St2, a disease-suppressive bacterium with antifungal activity toward the plant pathogen *Colletotrichum falcatum*. *Genome Announc* 2014; **2**: e01108-13.

31. Stanojkovic-Sebic A, Dinić Z, Ilicic R, Pivic R, Josic D. Effect of indigenous *Pseudomonas chlororaphis* strains on morphological and main chemical growth parameters of basil (*Ocimum basilicum* L.). *Ratar Povrt* 2017; **54**: 42–47.

32. Trögl J, Chauhan A, Ripp S, Layton AC, Kuncová G, Sayler GS. *Pseudomonas fluorescens* HK44: lessons learned from a model whole-cell bioreporter with a broad application history. *Sensors* 2012; **12**: 1544–1571.

33. Achouak W, Sutra L, Heulin T, Meyer JM, Fromin N, Degraeve S, et al.  *Pseudomonas brassicacearum* sp. nov. and  *Pseudomonas thivervalensis* sp. nov., two root-associated bacteria isolated from *Brassica napus* and  *Arabidopsis thaliana*. *Int J Syst Evol Microbiol* 2000; **50**: 9–18.

34. Nelkner J, Torres Tejerizo G, Hassa J, Lin TW, Witte J, Verwaaijen B, et al. Genetic potential of the biocontrol agent *Pseudomonas brassicacearum* (Formerly *P. trivialis*) 3Re2-7 unraveled by genome sequencing and mining, comparative genomics and transcriptomics. *Genes* 2019; **10**: 601–631.

35. Keel C, Weller DM, Natsch A, Défago G, Cook RJ, Thomashow LS. Conservation of the 2,4-diacetylphloroglucinol biosynthesis locus among fluorescent *Pseudomonas* strains from diverse geographic locations. *Appl Environ Microbiol* 1996; **62**: 552–563.

36. Raaijmakers JM, Weller DM. Natural plant protection by 2,4-diacetylphloroglucinol-producing *Pseudomonas* spp. in take-all decline soils. *Mol Plant Microbe Interact* 1998; **11**: 144–152.

37. Redondo-Nieto M, Barret M, Morrisey JP, Germaine K, Martinez-Granero F, Barahona E, et al. Genome sequence of the biocontrol strain *Pseudomonas fluorescens* F113. *J Bacteriol* 2012; **194**: 1273–1274.

38. Sikorski J, Stackebrandt E, Wackernagel W. *Pseudomonas kilonensis* sp. nov., a bacterium isolated from agricultural soil. *Int J Syst Evol Microbiol* 2001; **51**: 1549–1555.

39. Fuchs JG, Moënne-Loccoz Y, Défago G. The laboratory medium used to grow biocontrol *Pseudomonas* sp. Pf 153 influences its subsequent ability to protect cucumber from black root rot.

40. Catara V. Phenotypic and genomic evidence for the revision of *Pseudomonas corrugata* and proposal of *Pseudomonas mediterranea* sp. nov. *Int J Syst Evol Microbiol* 2002; **52**: 1749–1758.

41. Catara V. *Pseudomonas corrugata*: plant pathogen and/or biological resource? *Mol Plant Pathol* 2007; **8**: 233–244.

42. Alippi AM, López AC. First report of *Pseudomonas mediterranea* causing tomato pith necrosis in Argentina. *New Dis Rep* ; **20**: 34–34.

43. Scarlett CM, Fletcher JT, Roberts P, Lelliott RA. Tomato pith necrosis caused by *Pseudomonas corrugata* n. sp. *Ann Appl Biol* 1978; **88**: 105–114.

44. Catara V, Gardan L, Lopez MM. Phenotypic heterogeneity of *Pseudomonas corrugata* strains from southern Italy. *J Appl Microbiol* 1997; **83**: 576–586.

45. Strano CP, Bella P, Licciardello G, Caruso A, Catara V. Role of secondary metabolites in the biocontrol activity of *Pseudomonas corrugata* and *Pseudomonas mediterranea*. *Eur J Plant Pathol* 2017; **149**: 103–115.

46. Compeau G, Levy SB. Survival of rifampin-resistant Mutants of *Pseudomonas fluorescens* and *Pseudomonas putida* in soil systems. *Appl Environ Microbiol* 1988; **54**: 2432–2438.

47. Olcott MH, Henkels MD, Rosen KL, L.Walker F, Sneh B, Loper JE, et al. Lethality and developmental delay in *Drosophila melanogaster* larvae after ingestion of selected *Pseudomonas fluorescens* strains. *PLoS ONE* 2010; **5**: e12504.

48. Shinde S, Cumming JR, Collart FR, Noirot PH, Larsen PE. *Pseudomonas fluorescens* transportome is linked to strain-specific plant growth promotion in aspen seedlings under nutrient stress. *Front Plant Sci* 2017; **8**.

49. Barton MD, Petronio M, Giarrizzo JG, Bowling BV, Barton HA. The genome of *Pseudomonas fluorescens* strain R124 demonstrates phenotypic adaptation to the mineral environment. *J Bacteriol* 2013; **195**: 4793–4803.

50. Hong S, Lee C, Jang S-H. Purification and properties of an extracellular esterase from a cold-adapted *Pseudomonas mandelii*. *Biotechnol Lett* 2012; **34**: 1051–1055.

51. Jang S-H, Kim J, Kim J, Hong S, Lee C. Genome sequence of cold-adapted *Pseudomonas mandelii* strain JR-1. *J Bacteriol* 2012; **194**: 3263.

52. Dandie C, Burton D, Zebarth B, Trevors J, Goyer C. Analysis of denitrification genes and comparison of *nosZ*, *cnorB* and 16S rDNA from culturable denitrifying bacteria in potato cropping systems. *Syst Appl Microbiol* 2007; **30**: 128–138.

53. Formusa PA, Hsiang T, Habash MB, Lee H, Trevors JT. Genome Sequence of *Pseudomonas mandelii* PD30. *Genome Announc* 2014; **2**.

54. Duan J, Jiang W, Cheng Z, Heikkila JJ, Glick BR. The complete genome sequence of the plant growth-promoting bacterium *Pseudomonas* sp. UW4. *PloS One* 2013; **8**: e58640.

55. Dueholm MS, Petersen SV, Sønderkaer M, Larsen P, Christiansen G, Hein KL, et al. Functional amyloid in *Pseudomonas*. *Mol Microbiol* 2010; **77**: 1009–1020.

56. Dueholm MS, Danielsen HN, Nielsen PH. Complete genome sequence of *Pseudomonas* sp. UK4, a model organism for studies of functional amyloids in *Pseudomonas*. *Genome Announc* 2014; **2**: e00898-14.

57. Gaballa A, Abeysinghe PD, Urich G, Matthijs S, Greve HD, Cornelis P, et al. Trehalose induces antagonism towards *Pythium debaryanum* in *Pseudomonas fluorescens* ATCC 17400. *Appl Environ Microbiol* 1997; **63**: 4340–4345.

58. Deveau A, Palin B, Delaruelle C, Peter M, Kohler A, Pierrat JC, et al. The mycorrhiza helper *Pseudomonas fluorescens* BBc6R8 has a specific priming effect on the growth, morphology and gene expression of the ectomycorrhizal fungus *Laccaria bicolor* S238N. *New Phytol* 2007; **175**: 743–755.

59. Frey-Klett P, Pierrat JC, Garbaye J. Location and survival of mycorrhiza helper *Pseudomonas fluorescens* during establishment of ectomycorrhizal symbiosis between *Laccaria bicolor* and Douglas Fir. *Appl Environ Microbiol* 1997; **63**: 139–144.

60. Ohtsubo Y, Kishida K, Sato T, Tabata M, Kawasumi T, Ogura Y, et al. Complete genome sequence of *Pseudomonas* sp. strain TKP, isolated from a γ-hexachlorocyclohexane-degrading mixed culture. *Genome Announc* 2014; **2**: e01241-13.

61. Morales M, Sentchilo V, Bertelli C, Komljenovic A, Kryuchkova-Mostacci N, Bourdilloud A, et al. The genome of the toluene-degrading *Pseudomonas veronii* Strain 1YdBTEX2 and its differential gene expression in contaminated sand. *PLOS ONE* 2016; **11**: e0165850.

62. Rhodes ME. The Characterization of *Pseudomonas fluorescens*. *J Gen Microbiol* 1959; **21**: 221–263.

63. Demange P, Bateman A, Mertz C, Dell A, Piemont Y, Abdallah MA. Bacterial siderophores: structures of pyoverdins Pt, siderophores of *Pseudomonas tolaasii* NCPPB 2192, and pyoverdins Pf, siderophores of *Pseudomonas fluorescen*s CCM 2798. Identification of an unusual natural amino acid. *Biochemistry* 1990; **29**: 11041–11051.

64. Lemanceau P, Schippers’ B. Effect of pseudobactin 358 production by *Pseudomonas putida* WCS358 on suppression of  *Fusarium* wilt of carnations by nonpathogenic *Fusarium oxysporum* Fo47. *Appl Environ Microbiol* 1992; **58**: 2978–2982.

65. Zachow C, Tilcher R, Berg G. Sugar beet-associated bacterial and fungal communities show a high indigenous antagonistic potential against plant pathogens. *Microb Ecol* 2008; **55**: 119–129.

66. Zachow C, Jahanshah G, de Bruijn I, Song C, Ianni F, Pataj Z, et al. The novel lipopeptide poaeamide of the endophyte *Pseudomonas poae* RE*1-1-14 is involved in pathogen suppression and root colonization. *Mol Plant-Microbe Interact MPMI* 2015; **28**: 800–810.

67. Gardiner DM, Stiller J, Covarelli L, Lindeberg M, Shivas RG, Manners JM. Genomesequences of *Pseudomonas* spp. isolated from cereal crops. *Genome Announc* 2013; **1**: e00209-13.

68. Kluepfel DA. Involvement of root-colonizing bacteria in peach orchard soils suppressive of the nematode *Criconemella xenoplax*. *Phytopathology* 1993; **83**: 1240–1245.

69. Loper JE, Henkels MD, Rangel LI, Olcott MH, Walker FL, Bond KL, et al. Rhizoxin analogs, orfamide A and chitinase production contribute to the toxicity of *Pseudomonas protegens strain* Pf-5 to *Drosophila melanogaster*. *Environ Microbiol* 2016; **18**: 3509–3521.

70. Souza JT de, Boer M de, Waard P de, Beek TA van, Raaijmakers JM. Biochemical, genetic, and zoosporicidal properties of cyclic lipopeptide surfactants produced by *Pseudomonas fluorescens*. *Appl Environ Microbiol* 2003; **69**: 7161–7172.

71. Rhodes G, Bosma H, Studholme D, Arnold DL, Jackson RW, Pickup RW. The *rulB* gene of plasmid pWW0 is a hotspot for the site-specific insertion of integron-like elements found in the chromosomes of environmental *Pseudomonas fluorescens* group bacteria: Integron-like elements insert into *rulB* on plasmid pWW0. *Environ Microbiol* 2014; **16**: 2374–2388.

72. Fessehaie A, Walcott RR. Biological control to protect watermelon blossoms and seed from infection by *Acidovorax avenae* subsp. *citrulli*. *Phytopathology* 2005; **95**: 413–419.

73. Stockwell VO, Johnson KB, Sugar D, Loper JE. Control of fire blight by *Pseudomonas fluorescens* A506 and *Pantoea vagans* C9-1 applied as single strains and mixed inocula. *Phytopathology* 2010; **100**: 1330–1339.

74. Ghosh S, Qureshi A, Purohit H. Role of *Pseudomonas fluorescens* EGD-AQ6 biofilms in degrading elevated levels of p-hydroxybenzoate. *J Microb Biochem Technol* 2016.

75. Naseby DC, Way JA, Bainton NJ, Lynch JM. Biocontrol of *Pythium* in the pea rhizosphere by antifungal metabolite producing and non-producing *Pseudomona*s strains. *J Appl Microbiol* 2001; **90**: 421–429.

76. Meyer JB, Frapolli M, Keel C, Maurhofer M. Pyrroloquinoline quinone biosynthesis gene *pqqC*, a novel molecular marker for studying the phylogeny and diversity of phosphate-solubilizing *Pseudomonads*. *Appl Environ Microbiol* 2011; **77**: 7345–7354.

77. Stabler RA, Negus D, Pain A, Taylor PW. Draft genome sequences of *Pseudomonas fluorescens* BS2 and *Pusillimonas noertemannii* BS8, soil bacteria that cooperate to degrade the poly-γ-d-glutamic acid anthrax capsule. *Genome Announc* 2013; **1**: e00057-12.

78. Banowetz GM, Azevedo MD, Armstrong DJ, Halgren AB, Mills DI. Germination-Arrest Factor (GAF): biological properties of a novel, naturally-occurring herbicide produced by selected isolates of rhizosphere bacteria. *Biol Control* 2008; **46**: 380–390.

79. Elliott LF, Lynch JM. Plant growth-inhibitory pseudomonads colonizing winter wheat (*Triticum aestivum* L.) roots. *Plant Soil* 1985; **84**: 57–65.

80. Ruinelli M, Blom J, Pothier JF. Complete genome sequence of *Pseudomonas viridiflava* CFBP 1590, isolated from diseased cherry in France. *Genome Announc* 2017; **5**: e00662-17.

81. Young JM, Triggs CM. Evaluation of determinative tests for pathovars of *Pseudomonas syringae* van Hall 1902. *J Appl Bacteriol* 1994; **77**: 195–207.

82. Smee MR, Baltrus DA, Hendry TA. Entomopathogenicity to two hemipteran insects is common but variable across epiphytic *Pseudomonas syringae* strains. *Front Plant Sci* 2017; **8**: 2149.

83. Stavrinides J, McCloskey JK, Ochman H. Pea aphid as both host and vector for the phytopathogenic bacterium *Pseudomonas syringae*. *Appl Environ Microbiol* 2009; **75**: 2230–2235.

84. Vinatzer BA, Teitzel GM, Lee M-W, Jelenska J, Hotton S, Fairfax K, et al. The type III effector repertoire of *Pseudomonas syringae* pv. syringae B728a and its role in survival and disease on host and non-host plants. *Mol Microbiol* 2006; **62**: 26–44.

85. Bultreys A, Gheysen I. Biological and molecular detection of toxic lipodepsipeptide-producing *Pseudomonas syringae* strains and PCR identification in plants. *Appl Environ Microbiol* 1999; **65**: 19041909.

86. O’Brien HE, Thakur S, Gong Y, Fung P, Zhang J, Yuan L, et al. Extensive remodeling of the *Pseudomonas syringae* pv. avellanae type III secretome associated with two independent host shifts onto hazelnut. *BMC Microbiol* 2012; **12**: 141.

87. Janse JD. The bacterial disease of ash (*Fraxinus excelsior*), caused by *Pseudomonas syringae* subsp. savastanoi pv. fraxini III. Pathogenesis. *Eur J For Pathol* 1982; **12**: 218–231.

88. Psallidas, P.G. & Panagopoulos, Christos. A new bacteriosis of almond caused by *Pseudomonas amygdali* sp. nov. *Ann Inst Phytopathol Benaki* 1975; **11**: 94–108.

89. Thakur S, Weir BS, Guttman DS. Phytopathogen genome announcement: draft genome sequences of 62 *Pseudomonas syringae* type and pathotype strains. *Mol Plant Microbe Interact* 2016; **29**: 243–246.

90. Gardan L, Bollet C, Abu Ghorrah M, Grimont F, Grimont PAD. DNA Relatedness among the Pathovar Strains of *Pseudomonas syringae* subsp. savastanoi Janse (1982) and Proposal of *Pseudomonas savastanoi* sp. nov. *Int J Syst Bacteriol* 1992; **42**: 606–612.

91. Mazzaglia A, Studholme DJ, Taratufolo MC, Cai R, Almeida NF, Goodman T, et al. *Pseudomonas syringae* pv. actinidiae (PSA) Isolates from Recent Bacterial Canker of Kiwifruit Outbreaks Belong to the Same Genetic Lineage. *PLoS ONE* 2012; **7**: e36518.

92. Takikawa Y, Serizawa S, Ichikawa T, Tsuyumu S, Goto M. *Pseudomonas syringae* pv. actinidiae pv. nov.: the causal bacterium of canker of kiwifruit in Japan. *Jpn J Phytopathol* 1989; **55**: 437–444.

93. Buell CR, Joardar V, Lindeberg M, Selengut J, Paulsen IT, Gwinn ML, et al. The complete genome sequence of the Arabidopsis and tomato pathogen *Pseudomonas syringae* pv. tomato DC3000. *Proc Natl Acad Sci* 2003; **100**: 10181–10186.

94. Cuppels DA. Generation and Characterization of Tn5 Insertion Mutations in *Pseudomonas syringae* pv. tomato. *Appl Environ Microbiol* 1986; **51**: 323.

95. Vallet-Gely I, Novikov A, Augusto L, Liehl P, Bolbach G, Pechy-Tarr M, et al. Association of hemolytic activity of *Pseudomonas entomophila*, a versatile soil bacterium, with cyclic lipopeptide production. *Appl Environ Microbiol* 2010; **76**: 910–921.

96. Vodovar N, Vinals M, Liehl P, Basset A, Degrouard J, Spellman P, et al. *Drosophila* host defense after oral infection by an entomopathogenic *Pseudomonas* species. *Proc Natl Acad Sci* 2005; **102**: 11414–11419.

97. Geels FP, Schippers B. Selection of antagonistic fluorescent *Pseudomonas* spp. and their root colonization and persistence following treatment of seed potatoes. *J Phytopathol* 1983; **108**: 193–206.

98. Meziane H, Van Der Sluis I, Van Loon LC, Höfte M, Bakker PAHM. Determinants of *Pseudomonas putida* WCS358 involved in inducing systemic resistance in plants. *Mol Plant Pathol* 2005; **6**: 177–185.

99. Fernández M, Porcel M, de la Torre J, Molina-Henares MA, Daddaoua A, Llamas MA, et al. Analysis of the pathogenic potential of nosocomial *Pseudomonas putida* strains. *Front Microbiol* 2015; **6**.

100. Gupta IR, Anderson AJ, Rai M. Toxicity of fungal-generated silver nanoparticles to soil-inhabiting *Pseudomonas putida* KT2440, a rhizospheric bacterium responsible for plant protection and bioremediation. *J Hazard Mater* 2015; **286**: 48–54.

101. Planchamp C, Glauser G, Mauch-Mani B. Root inoculation with *Pseudomonas putida* KT2440 induces transcriptional and metabolic changes and systemic resistance in maize plants. *Front Plant Sci* 2015; **5**.

102. Validov SZ, Kamilova F, Lugtenberg BJJ. *Pseudomonas putida* strain PCL1760 controls tomato foot and root rot in stonewool under industrial conditions in a certified greenhouse. *Biol Control* 2009; **48**: 6–11.

103. van Belkum A, Soriaga LB, LaFave MC, Akella S, Veyrieras J-B, Barbu EM, et al. Phylogenetic Distribution of CRISPR-Cas Systems in Antibiotic-Resistant *Pseudomonas aeruginosa*. *mBio* 2015; **6**: e01796-15.

104. Sood U, Hira P, Kumar R, Bajaj A, Rao DLN, Lal R, et al. Comparative genomic analyses reveal core-genome-wide genes under positive selection and major regulatory hubs in outlier strains of *Pseudomonas aeruginosa*. *Front Microbiol* 2019; **10**: 53.

105. Basso P, Wallet P, Elsen S, Soleilhac E, Henry T, Faudry E, et al. Multiple *Pseudomonas* species secrete exolysin‐like toxins and provoke Caspase‐1‐dependent macrophage death. *Environ Microbiol* 2017; **19**: 4045–4064.

106. Elsen S, Huber P, Bouillot S, Couté Y, Fournier P, Dubois Y, et al. A type III secretion negative clinical strain of *Pseudomonas aeruginosa* employs a two-partner secreted exolysin to induce hemorrhagic pneumonia. *Cell Host Microbe* 2014; **15**: 164–176.

107. Roy PH, Tetu SG, Larouche A, Elbourne L, Tremblay S, Ren Q, et al. Complete genome sequence of the multiresistant taxonomic outlier *Pseudomonas aeruginosa* PA7. *PloS One* 2010; **5**: e8842.

108. Haynes WC. *Pseudomonas aeruginosa* - its characterization and identification. *J Gen Microbiol* 1951; **5**: 939–950.

109. Kohler W. Zur Serologie der *Pseudomonas aeruginosa*. *Z Immunforsch Exp Ther* 1957; **114**: 282.

110. Kos VN, Déraspe M, McLaughlin RE, Whiteaker JD, Roy PH, Alm RA, et al. The resistome of *Pseudomonas aeruginosa* in relationship to phenotypic susceptibility. *Antimicrob Agents Chemother* 2015; **59**: 427–436.

111. Lau GW, Goumnerov BC, Walendziewicz CL, Hewitson J, Xiao W, Mahajan-Miklos S, et al. The *Drosophila melanogaster* Toll pathway participates in resistance to infection by the Gram-negative human pathogen *Pseudomonas aeruginosa*. *Infect Immun* 2003; **71**: 4059–4066.

112. Mahajan-Miklos S, Tan M-W, Rahme LG, Ausubel FM. Molecular mechanisms of bacterial virulence elucidated using a *Pseudomonas aeruginosa*– *Caenorhabditis elegans* pathogenesis model. *Cell* 1999; **96**: 47–56.

113. Starkey M, Rahme LG. Modeling *Pseudomonas aeruginosa* pathogenesis in plant hosts. *Nat Protoc* 2009; **4**: 117–124.

114. Walker TS, Bais HP, Déziel E, Schweizer HP, Rahme LG, Fall R, et al. *Pseudomonas aeruginosa* - plant root interactions. Pathogenicity, biofilm formation, and root exudation. *Plant Physiol* 2004; **134**: 320–331.

115. Nakano K, Terabayashi Y, Shiroma A, Shimoji M, Tamotsu H, Ashimine N, et al. First complete genome sequence of *Pseudomonas aeruginosa* (Schroeter 1872) Migula 1900 (DSM 50071 ^T^ ), determined using PacBio Single-Molecule Real-Time technology. *Genome Announc* 2015; **3**: e00932-15.

116. van der Zee A, Kraak WB, Burggraaf A, Goessens WHF, Pirovano W, Ossewaarde JM, et al. Spread of carbapenem resistance by transposition and conjugation among *Pseudomonas aeruginosa*. *Front Microbiol* 2018; **9**: 2057.

117. Boyle B, Fernandez L, Laroche J, Kukavica-Ibrulj I, Mendes CMF, Hancock RW, et al. Complete genome sequences of three *Pseudomonas aeruginosa* isolates with phenotypes of polymyxin B adaptation and inducible resistance. *J Bacteriol* 2012; **194**: 529–530.

118. Li G, Shen M, Le S, Tan Y, Li M, Zhao X, et al. Genomic analyses of multidrug resistant *Pseudomonas aeruginosa PA1* resequenced by single-molecule real-time sequencing. *Biosci Rep* 2016; **36**: e00418.

119. Maciel-Vergara G, Jensen AB, Eilenberg J. Cannibalism as a possible entry route for opportunistic pathogenic bacteria to insect hosts, exemplified by *Pseudomonas aeruginosa*, a pathogen of the giant mealworm *Zophobas morio*. *Insects* 2018; **9**: 88–103.

120. Syazni, Yanagisawa M, Kasuu M, Nakasaki K, Ariga O. Draft genome sequence of the nonmarine agarolytic bacterium *Cellvibrio* sp. OA-2007. *Genome Announc* 2015; **3**: e00468-15.

121. Wu Y-R, He J. Characterization of a xylanase-producing *Cellvibrio mixtus* strain J3-8 and its genome analysis. *Sci Rep* 2015; **5**: 10521.

122. Humphry DR. Reclassification of *Pseudomonas fluorescens* subsp. cellulosa NCIMB 10462 (Ueda et al. 1952) as *Cellvibrio japonicus* sp. nov. and revival of *Cellvibrio vulgaris* sp. nov., nom. rev. and *Cellvibrio fulvus* sp. nov., nom. rev. *Int J Syst Evol Microbiol* 2003; **53**: 393–400.

123. Bergmark L, Poulsen PHB, Al-Soud WA, Norman A, Hansen LH, Sørensen SJ. Assessment of the specificity of *Burkholderia* and *Pseudomonas* qPCR assays for detection of these genera in soil using 454 pyrosequencing. *FEMS Microbiol Lett* 2012; **333**: 77–84.
